# The minimum crystal size needed for a complete diffraction data set

**DOI:** 10.1107/S0907444910007262

**Published:** 2010-03-24

**Authors:** James M. Holton, Kenneth A. Frankel

**Affiliations:** a Department of Biochemistry and Biophysics, University of California, San Francisco, CA 94158-2330, USA; b Lawrence Berkeley National Laboratory, Berkeley, CA 94720, USA

**Keywords:** radiation damage, minimum crystal size, protein macromolecular crystallography, scattering power

## Abstract

In this work, classic intensity formulae were united with an empirical spot-fading model in order to calculate the diameter of a spherical crystal that will scatter the required number of photons per spot at a desired resolution over the radiation-damage-limited lifetime. The influences of molecular weight, solvent content, Wilson *B* factor, X-ray wavelength and attenuation on scattering power and dose were all included. Taking the net photon count in a spot as the only source of noise, a complete data set with a signal-to-noise ratio of 2 at 2 Å resolution was predicted to be attainable from a perfect lysozyme crystal sphere 1.2 µm in diameter and two different models of photoelectron escape reduced this to 0.5 or 0.34 µm. These represent 15-fold to 700-fold less scattering power than the smallest experimentally determined crystal size to date, but the gap was shown to be consistent with the background scattering level of the relevant experiment. These results suggest that reduction of background photons and diffraction spot size on the detector are the principal paths to improving crystallographic data quality beyond current limits.

## Abstract 2

A formula for absolute scattering power is derived to include spot fading arising from radiation damage and the crystal volume needed to collect diffraction data to a given resolution is calculated.

## 1. Introduction

The last 15 years have seen many experimental estimates of how small a protein crystal can be and still yield a complete data set (Gonzalez & Nave, 1994 ▶; Glaeser *et al.*, 2000 ▶; Teng & Moffat, 2000 ▶, 2002 ▶; Facciotti *et al.*, 2003 ▶; Sliz *et al.*, 2003 ▶; Li *et al.*, 2004 ▶; Nelson *et al.*, 2005 ▶; Sawaya *et al.*, 2007 ▶; Coulibaly *et al.*, 2007 ▶; Standfuss *et al.*, 2007 ▶; Moukhametzianov *et al.*, 2008 ▶; reviewed by Holton, 2009 ▶) and this size has been decreasing as technology improves. But is there a theoretical limit? The work presented here establishes a firm theoretical framework for computing the absolute signal available from very small macromolecular crystals and every effort is made to explicitly and unambiguously spell out the definitions and derivations. The *International Tables for Crystallography* (Wilson & Prince, 1999 ▶) contain most of the critical pieces of the puzzle assembled here and the original references are spread out over nearly a century of literature.

Here, we endeavor to keep the theory general and independent of the limitations of current diffraction hardware. For example, the time-honored practice of recording the three-dimensional diffraction pattern on as few images as possible was not simply an effort to save money on film, but to minimize noise intrinsic to the detection process such as ‘fog’ on the film or the read-out circuit of a charge-coupled device (CCD). Counting detectors such as multi-wire (Cork *et al.*, 1974 ▶) and pixel arrays (Kraft *et al.*, 2009 ▶) do not have this kind of noise and the optimal data-collection strategy with these detectors is different (Xuong *et al.*, 1985 ▶; Schulze-Briese *et al.*, 2007 ▶). For simplicity, in the present work we consider the X-­ray detector and indeed the entire diffractometer to be an ideal device subject only to the shot noise of the net spot photons themselves (the square root of the number of counts). All other sources of noise, including background scattering, are neglected until the discussion in §[Sec sec3.2]3.2.

The formula for the integrated intensity of a spot was introduced by Darwin (1914 ▶), but much subsequent work was required to fill out the original theory. For example, Darwin’s variable ‘*f*’ required the development of quantum theory to explain its observed value (Debye, 1915 ▶, 1988 ▶). The resulting orbital shapes (Slater, 1929 ▶) led directly to the cross-sections needed to compute absorption effects in the 1960s and steady improvements continue to this day (Hubbell, 2006 ▶). Only recently has it become clearly established that radiation damage at cryogenic temperatures is proportional to dose (Henderson, 1990 ▶; Gonzalez & Nave, 1994 ▶; Glaeser *et al.*, 2000 ▶; Sliz *et al.*, 2003 ▶; Leiros *et al.*, 2006 ▶; Owen *et al.*, 2006 ▶; Garman & McSweeney, 2007 ▶; Garman & Nave, 2009 ▶; Holton, 2009 ▶) and this understanding enabled the present work.

The intensity of a Bragg spot is not simply the square of the structure factor, but depends on several other factors including exposure time, crystal volume and the geometry of diffraction. Consequently, the absolute number of photons in a spot (which determines the maximum possible signal-to-noise ratio) depends on exactly where the spot falls on the detector surface. Algorithms for computing these intensity ‘correction’ factors are encoded into most data-processing programs, but the source codes are not always available and in many cases the implemented corrections only apply to particular camera geometries. Therefore, the reproducibility and generality of the results presented here requires a clear description of each correction factor and we begin by defining the relevant co­ordinate system.

## 2. Methods

### 2.1. Coordinate system

**Figure 1:**
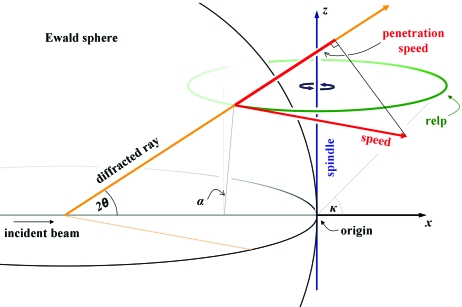
Coordinate system. The *x* axis is occupied by the X-ray beam and the spindle rotates the crystal (at the origin) about the *z* axis. The *y* axis is not shown as it is very nearly perpendicular to the page. The reciprocal-lattice point (relp) of interest is described here by the circle it traces out as the crystal is rotated. Note that it intersects the Ewald sphere twice and that the ‘penetration speed’ is the component of the relp’s velocity that is perpendicular to the Ewald sphere surface. The ratio of the ‘actual speed’ to the ‘penetration speed’ is the Lorentz factor. The diffracted ray passes through the point of intersection, but evolves from the center of the Ewald sphere (not the origin!), which is an unfortunate conceptual flaw in Ewald’s construction. Nevertheless, the take-off angle (2θ) obtained is the same as that observed in real space. The angles α and κ used in (3) and Appendix *C* are shown.

There are many possible ways to assign *xyz* coordinates to a diffractometer; unfortunately, most of them have been employed at one time or another and few data-processing programs share exactly the same convention. Here, we will adopt a ‘classic’ coordinate system essentially identical to that described in chapter 7 of Arndt & Wonacott (1977 ▶), which is also the coordinate system used by the data-processing program *MOSFLM* (Leslie, 2006 ▶). In this system, *x* is the direction of the X-ray beam, *z* is the (horizontal) spindle axis and *y* is ‘up’ (opposing gravity) or perpendicular to the page in Fig. 1 ▶.

### 2.2. Spot intensity

Typically, crystallographic data-processing and model-refinement programs assign an arbitrary ‘scale factor’ for the observed spot intensities to put them on the same scale as the structure factors calculated from the model, but the exact relationship between the intensity of a fully recorded spot and the square of the structure factor is given by Darwin’s formula (Darwin, 1914 ▶, 1922 ▶; Blundell & Johnson, 1976 ▶) and instructive re-derivations can be found in textbooks by James (1962 ▶) and Woolfson (1997 ▶),
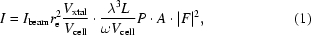
where *I* is the integrated spot intensity (photons/spot), *I*_beam_ is the intensity of the incident beam (photons s^−1^ m^−2^), *r*_e_ is the classical electron radius (2.818 × 10^−15^ m), *V*_xtal_ is the illuminated volume of the crystal (in m^3^), *V*_cell_ is the volume of the crystal unit cell (in m^3^), λ is the X-ray wavelength (in m), ω is the angular velocity of the crystal (radians s^−1^; §[Sec sec2.8]2.8), *L* is the Lorentz factor (speed/speed; §[Sec sec2.3]2.3), *P* is the polarization factor (photons/photons; §[Sec sec2.4]2.4), *A* is the X-ray transmittance of the path through the crystal to the spot (photons/photons; §2.5) and *F* is the structure factor of the unit cell at the relp of interest (electron equivalents; §2.7).

The abbreviation ‘relp’ (reciprocal-lattice point) is used to denote a particular point in reciprocal space, distinct from its symmetry mates (Ramachandran & Wooster, 1951 ▶; Helliwell, 1999 ▶), and here we use ‘spot’ to refer to a single observation of a relp and ‘*hkl*’ to indicate the sum of all symmetry-equivalent spots (merging anomalous pairs). Note that all quantities entered into (1) are in metre–kilogram–second (MKS) units, including the X-ray wavelength (λ), and that the units of ‘intensity’ for spots (photons/spot) are not the same as those for either the incident beam (photons s^−1^ m^−2^) or classical electron scattering (photons sr^−1^). Despite this, all of these quantities remain commonly referred to as ‘intensity’, leading to a considerable amount of confusion if the units are not given explicitly. The change of units arises because the full spot intensity (photons/spot) is obtained by integrating over the relp as it moves through the Ewald sphere (Ewald, 1913 ▶; Arndt & Wonacott, 1977 ▶; Helliwell, 1999 ▶) and therefore several geometric factors must be taken into account.

Experimental confirmation of Darwin’s formula has been presented by Moseley & Darwin (1913 ▶), Bragg *et al.* (1921*a* ▶,*b* ▶, 1922 ▶), Compton & Freeman (1922 ▶) and many others since. For an example calculation using (1), consider a 100 µm diameter spherical protein crystal with all three unit-cell edges 50 Å long. Assume that for a particular relp at 2 Å resolution we have *F* = 170 electron equivalents (see §[Sec sec2.7]2.7) and further assume some crystal orientation that assigns *L* = 2.2, *P* = 0.92 and *A* = 96% to this relp (see §§[Sec sec2.3]2.3, [Sec sec2.4]2.4 and [Sec sec2.5]2.5, respectively). If the crystal rotates at 1° s^−1^ in a uniform beam of 1 Å X-rays with 10^12^ photons s^−1^ passing into the 100 µm diameter circular cross-section of the crystal, then (1) predicts an integrated full spot intensity of 109 011 photons. This calculation was found to be in remarkable agreement with experimentally observed spot intensities from a lysozyme crystal (not shown) on the protein crystallography beamline 8.3.1 at the Advanced Light Source (instrument described by MacDowell *et al.*, 2004 ▶). Once *I*_beam_ had been calibrated (Owen *et al.*, 2009 ▶), the discrepancy between calculation and experiment was essentially the uncertainty in our visual estimate of *V*_xtal_ (about 15%).

The flux density *I*_beam_ is a constant in (1), which implies that the crystal is ‘bathed’ in a ‘flat-top’ or ‘top-hat’ beam. Real X-­ray beams are seldom this perfect, but any crystal in any beam may be formally broken up into tiny cubes small enough for *I*_beam_ to be considered constant over each cube and the total spot intensity obtained by summing the results of (1) for all the cubes. However, if *I*_beam_ is the same for every cube there is clearly no need to break up the crystal; conversely, if the crystal has constant thickness along the beam direction then the average flux density experienced by the crystal (regardless of beam shape) may be used as *I*_beam_ in (1). Only if both the crystal shape and the beam profile have irregular shapes does (1) need to be integrated over the beam profile and crystal volume. However, we show in §[Sec sec2.11]2.11 and Appendix *C* (deposited as supplementary material^1^) that the damage-limited spot intensity is independent of *I*_beam_, obviating the need to consider beam and crystal shapes, so for simplicity in this work we will consider a spherical crystal ‘bathed’ in a top-hat beam.

Note that (1) does not depend on the mosaic structure of the crystal and indeed a crystal consisting of a single mosaic domain or thousands of mosaic domains will still yield exactly the same integrated spot intensity (*I*) as long as the mosaic domains are small when compared with the attenuation depth (μ^−1^) of the X-rays in the crystal. This depth is typically several millimetres for 1 Å X-rays (see the end of §[Sec sec2.5]2.5) and protein crystals this large are very rare, let alone single-domain crystals (Snell *et al.*, 2003 ▶). A common misconception that protein microcrystals consisting of a single mosaic domain will produce more intense spots than expected from Darwin’s formula seems to have arisen from the above-mentioned confusion over the several possible meanings of the word ‘intensity’ (discussed further in §[Sec sec2.7]2.7). In truth, however, (1) was derived for small and single-domain crystals and also applies to the ‘ideally imperfect’ case of a large crystal with many mosaic domains (Darwin, 1922 ▶). Large single-domain crystals that approach the length scale of the attenuation depth of the X-rays actually produce weaker spots than predicted by (1) owing to extinction effects (James, 1962 ▶; Woolfson, 1997 ▶; Sabine, 1999 ▶; Authier, 2004 ▶).

### 2.3. Lorentz factor

The Lorentz factor *L* in (1) is always greater than one and is the ratio of the speed of a rotating relp to the ‘penetration speed’ at which it transits the Ewald sphere (Fig. 1 ▶). This Lorentz factor in crystallography^2^ is not to be confused with its inverse, the Lorentz correction *L* ^−1^ which data-processing programs such as *MOSFLM* (Leslie, 2006 ▶) use to ‘correct’ for this effect by multiplying observed integrated intensities by *L* ^−1^. The description of the Lorentz factor in *International Tables for Crystallography* (Lipson & Langford, 1999 ▶) notes that some confusion has arisen over the definition of the Lorentz factor because Lorentz never published it. Instead, it seems he wrote a letter to Debye, who included it as a second note added in proof (Debye, 1914 ▶, 1988 ▶).

Essentially, the Lorentz factor accounts for how the integrated intensity (photons/spot) of a relp will be higher if it moves slowly through the Bragg condition than if it moves quickly. Indeed, the angular velocity of the crystal (ω) divided by the Lorentz factor (*L*) is the angular velocity of the relp as ‘seen’ from the origin (see Fig. 1 ▶). This geometric correction is therefore grouped with other geometric factors in (1) such as ω. The cube of the wavelength (λ^3^) and one of the unit-cell volume (*V*_cell_) terms are also geometric corrections since these are involved in the size of the integration volume in reciprocal space (chapter 6 of Woolfson, 1997 ▶).

It is instructive to consider the relationship between the Lorentz factor and the spot position on the detector. This will obviously depend on the camera geometry, but in the common case in which the crystal rotation axis is perpendicular to the X-­ray beam the Lorentz factor (*L*) is given by
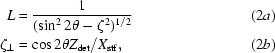
where θ is the Bragg angle, ζ (λd*·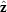) is a normalized projection of the relp vector onto the rotation axis (*z*), ζ_⊥_ is ζ in terms of spot coordinates on a flat detector normal to the incident beam, *Z*_det_ is the coordinate of the diffraction spot on the detector along the axis parallel to the rotation axis (relative to the beam center in mm) and *X*_stf_ is the sample-to-detector distance along the direct-beam path (in mm).

The Bragg angle θ is defined as half of the angle between the direct-beam path and the diffracted ray (see Fig. 1 ▶). Any given relp can be represented as a vector d* that will always have length d* = 1/*d*, where *d* is the *d*-spacing (in Å) of the spot. No matter how the crystal is rotated, the *d*-spacing of a spot does not change. The polar coordinate ζ (Helliwell, 1999 ▶) is calculated by taking the *z* component of d* (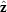 is the unit vector along the *z* axis) and multiplying it by the X-ray wavelength λ (in Å). This is because the *z* component of d* has dimensions of Å^−1^ and ζ must be dimensionless to be meaningfully related to sinθ.

In the also common case in which the detector is a flat plane and normal to the incident X-ray beam ζ may be conveniently replaced with ζ_⊥_ from (2*b*). However, moving the detector does not change the *L* of a given relp and ζ_⊥_ serves simply as a convenient way to map the Lorentz factor onto the detector face. For arbitrary detector positions ζ must be computed from the spindle geometry and in the general case of the beam not being perfectly normal to the rotation axis *L* must be calculated by taking the projection of the relp velocity vector along the diffracted ray (as shown in Fig. 1 ▶).

Arbitrary rotations of the crystal will rotate the vector d* by exactly the same angles and if the crystal is oriented such that d* approaches the spindle axis (*z* axis) it will eventually cross into a ‘blind region’ (Arndt & Wonacott, 1977 ▶; Helliwell, 1999 ▶) where spindle rotation alone cannot bring the relp onto the Ewald sphere. As the relp approaches this blind region the denominator of (2*a*) becomes smaller and smaller and the Lorentz factor approaches infinity. Crossing into the blind region, the quantity under the square root in (2*a*) becomes zero or less and the Lorentz factor becomes undefined.

It is important to note, however, that an infinite Lorentz factor does not actually imply an infinite spot intensity. This is because the relps are not infinitely sharp points, but rather occupy a volume in reciprocal space that must pass completely through the Ewald sphere for (1) to be valid. In fact, the size and shape of this reciprocal-space volume is simply the Fourier transform of the size and shape of the mosaic domain producing it, but a detailed discussion of spot shapes is beyond the scope of this work. It will suffice here to say that the blind region is effectively enlarged by an angle comparable to the crystal mosaic spread, ‘swallowing’ the infinite Lorentz factors. The few spots that are close to the rotation axis will indeed have very large Lorentz factors, but also a very wide angular range of reflection (rocking width), so on a typical diffraction image these high-*L* spots are roughly the same intensity (photons/spot) as any other. A discussion of rotation range will continue in §[Sec sec2.8]2.8.

### 2.4. Polarization factor

The polarization factor *P* is always less than one and accounts for losses of scattering efficiency when the incident-beam and scattered-beam E-vectors do not line up. That is, the E-vector of any electromagnetic wave must always be perpendicular to the direction of travel (Maxwell, 1865 ▶; Purcell, 1985 ▶), but the direction of travel changes upon scattering. *P* is simply the dot product of the E-vectors of the incident and scattered waves (averaged over all incident E-­vectors) and here we use the convenient expression given by Drenth (1999 ▶) (Azároff, 1955 ▶; Kahn *et al.*, 1982 ▶),
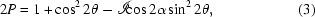
where *P* is the polarization factor used in (1) (photons/photons), θ is the Bragg angle, α is the angle between the projections of the *z* axis and the diffracted ray onto a plane normal to the incident beam and 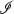 is the degree of polarization.

Note that the polarization factor *P* varies from spot to spot whereas 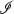 is the ‘polarization’ entered into most diffraction data-processing programs. 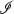 ranges from 1 to 0 to −1 as the incident E-vector varies from ‘horizontal’ (along the *z* axis) to un­polarized to ‘vertical’, respectively. The ‘plane normal to the incident beam’ invoked to define α here is any plane parallel to both the *y* and *z* axes (see α in Fig. 1 ▶ as well as Arndt & Wonacott, 1977 ▶).

Many synchrotron-based diffractometers are designed with horizontal spindle axes (as defined here) because in this geometry the strong horizontal polarization of synchrotron radiation (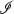 close to 1) tends to cancel the Lorentz factor and the ‘hole’ in scattering owing to polarization at 2θ = 90° and α = 0° coincides with the blind region (§[Sec sec2.3]2.3). However, the average value of the product *LP* is independent of 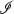 (see §[Sec sec2.6]2.6) and therefore spindle orientation has no effect on average intensity (photons/spot) in a given resolution bin. The only practical concern is that many data-processing programs throw out spots with large *L* because such spots are very sensitive to small errors in crystal orientation, but even when *L* > 5 spots are rejected the ‘penalty’ of a vertical spindle (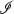 = −1) in the 2 Å bin using 1 Å radiation is only a 10% drop in photons/*hkl* (not shown). Indeed, for such data *P* ranges from 1 to 0.77 and this variation diminishes further as the pattern is compressed into lower angles at shorter wavelengths because (3) depends purely on the geometry of the camera and not on the X-ray wavelength used. The mechanical stability advantages of a vertical spindle for small crystals therefore come at only a marginal cost to photons/spot.

### 2.5. Sample attenuation

The attenuation factor *A* in (1) is an average optical transmittance and is always less than one. For full accuracy, photons from each point in the X-ray source must be ray-traced to every accessible part of the crystal volume and from there out into the spot. The transmittance along each path depends on the size, shape and atomic composition of the crystal and any other substances it traverses (including air). The profile of the beam acts as a ‘weighting function’ and *A* is the average transmittance over all possible paths. Given the potential complexity of the shapes involved, the only general expression for *A* is the triple integral
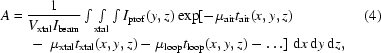
where *A* is the attenuation factor (photons/photons), *V*_xtal_ is the volume of the crystal (m^3^), *I*_beam_ is the total intensity of the incident beam (photons s^−1^ m^−2^), *I*_prof_ is the intensity of the beam profile at the coordinate 0, *y*, *z* (photons s^−1^ m^−2^), μ_*x*_ is the attenuation coefficient of substance *x*, μ_*x*_^−1^ is the attenuation length (m) and *t*_*x*_ is the component of the total path taken by X-rays through substance *x via* crystal coordinate *x*, *y*, *z* (m).

The complexity arises because the scattering and attenuation processes must be co-integrated over the illuminated volume of the crystal (*V*_xtal_). The path taken by the incident beam is only important up to the point location of the ‘scattering event’ and from there the materials between the scattering event and the location of the diffraction spot must be considered. This integral can be solved analytically for the simple case of a flat slab-shaped crystal with uniform μ and the formula for this solution is presented in *International Tables for Crystallography* (Maslen, 1999 ▶). However, for anything other than a flat slab there is no analytic solution for (4) and even a perfect sphere must be evaluated numerically. Nevertheless, a sphere is a convenient ‘average shape’ for a protein crystal and look-up tables are available for this integral (Dwiggins, 1975 ▶; Flack & Vincent, 1978 ▶; Maslen, 1999 ▶). For the calculation at hand, we consider a spherical crystal of radius *R* with uniform attenuation coefficient μ_xtal_ in a uniform ‘flat-top’ beam and denote the total transmission of a beam diffracting at angle 2*θ* simply as 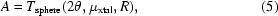
where *A* is the attenuation factor (photons/photons), *T*_sphere_ is the numerical solution to (4) for a sphere in a vacuum, 2θ is the angle between the incident and diffracted beams, μ_xtal_ is the attenuation coefficient of the crystal (m^−1^) and *R* is the radius of the spherical crystal (m).

The value of μ for each substance is obtained using its density (ρ) and the tabulated X-ray cross-sections (Storm & Israel, 1970 ▶; Berger & Hubbell, 1987 ▶; Creagh & Helliwell, 1999 ▶) of the chemical elements comprising it (reviewed by Hubbell, 2006 ▶). A convenient program for the accurate calculation of μ for a particular protein crystal is *RADDOSE* (Murray *et al.*, 2004 ▶; Paithankar *et al.*, 2009 ▶); for the calculations presented here we use an average empirical formula for protein, H_49.8_C_31.8_N_8.56_O_9.54_S_0.249_, determined from a survey (not shown) of the Protein Data Bank (Berman *et al.*, 2002 ▶). Taking 1 Å X-rays, for example, the values for μ in protein, water and the 50% solvent protein crystal used in this work are 2.78, 2.85 and 2.81 cm^−1^, respectively. This yields an attenuation depth μ^−1^_xtal_ of 3.6 mm, so a 2.5 mm thick protein crystal is required to reduce a spot intensity (photons/spot) by half and a 100 µm crystal reduces no spot intensity by more than ∼2.7%. Therefore, *A* is a small correction in typical cases and only becomes significant if strongly absorbing atoms are soaked into the crystal (see Holton, 2009 ▶) or if long-wavelength X-rays are used. For example, at the S *K* edge (5 Å wavelength) μ^−1^_xtal_ ≃ 32 µm and attenuation can reduce the spot intensities from a 100 µm crystal by as much as 96% (*A* = 0.04).

### 2.6. Average Lorentz–polarization factor and completeness

Since we are concerned here with the average value of a spot intensity (photons/spot) at a given resolution, we must know the average value of the product of the Lorentz and polarization factors (*LP*). It is also important to account for relps that fall into the ‘blind region’ (§[Sec sec2.3]2.3) as these will not contribute to the merged signal of an *hkl* index at one wavelength but may contribute at another. The fraction of all relps in a given resolution bin that can be observed by rotating about a single axis (f_obs_) is simply cosθ (see Appendix *A*) and if we average the product of (2*a*) and (3) for these accessible relps (Appendix *B*) we obtain the exact expressions
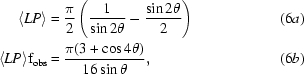
where f_obs_ is the fraction of relps at this resolution that will cross the Ewald sphere using a single axis (cosθ) and θ is the Bragg angle. Note the use of angle brackets 〈〉 to denote average values and that 〈*LP*〉 and f_obs_ depend only on the Bragg angle (θ) and thus are independent of wavelength (λ) and the degree of polarization 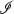 from (3). However, as Bragg’s law relates λ to θ, 〈*LP*〉f_obs_ tends to cancel one of the λ terms in (1), but not exactly.

### 2.7. Average structure factor

The ‘structure factor’ has been defined (Debye & Scherrer, 1918 ▶; Hartree, 1925 ▶; Coppens, 1999 ▶) as the ratio of the amplitude of an electromagnetic wave scattered by an object of interest to that scattered by a single classical electron (Thomson, 1906 ▶; chapter 2 of Woolfson, 1997 ▶; Maslen *et al.*, 1999*a* ▶), hence Thomson’s classical electron cross-section (*r*_e_^2^) is included in (1). The *F* in (1) is the structure factor of one unit cell, which must be isolated in space for the intensity (photons sr^−1^) to be computed directly from *F*. The other terms in (1) represent the ratio of the intensity scattered from a single unit cell to that of the entire crystal.

The apparent amplification from one *V*_cell_ term in (1) is effectively cancelled by the average square structure factor 〈*F* ^2^〉, which is proportional to *V*_cell_ when the number of atoms per unit volume is fixed. This cancellation arises because the average scattering from a macromolecule at *d*-spacings better than ∼4 Å is essentially the same as that of a random distribution of atoms (Wilson, 1942 ▶, 1949 ▶; Shmueli & Wilson, 1999 ▶) and the total structure factor of a random arrangement of atoms rapidly approaches the structure factor of one atom (*f*_a_) multiplied by the square root of the number of atoms. That is, when the scattered waves from a group of atoms are in no way ‘correlated’ with each other, the total scattered intensity (photons s^−1^ sr^−1^) is the sum of the intensities that would be seen from individual atoms and the square root of this total intensity is (by definition) proportional to the structure factor of the group. Conversely, if the atomic positions are perfectly correlated (such as in a regular lattice) then the amplitudes add in a nonrandom way and the intensity scattered in some directions (diffraction spots) becomes proportional to the square of the number of atoms. It is important to remember that this intensity has units of photons s^−1^ sr^−1^, where steradians (sr) are the units of solid angle. For example, 10^6^ photons s^−1^ emitted in completely random directions are described by an ‘intensity’ of 10^6^/4π = 79 577 photons s^−1^ sr^−1^ and a square detector pixel 100 µm in size and 100 mm from the sample (10^−6^ sr) will intercept about one photon every 12.6 s. Although the intensity (photons s^−1^ sr^−1^) scattered by a crystal of *N* atoms can be very large, this is only true over a very small solid angle and as the size of the crystal (or mosaic domain) increases this solid angle becomes proportionally smaller. In general, this patch of high intensity is much smaller than a pixel, but the observed intensity (in photons) is given by the integral of photons s^−1^ sr^−1^ over the entire pixel and rocking width of the relp (chapters 2 and 6 of Woolfson, 1997 ▶). The change in units whilst using the same word ‘intensity’ has historically led to some confusion, no doubt arising in part from Darwin’s formula appearing more than half a century before the first use of the word ‘pixel’ in the scientific literature.

It is instructive here to examine how the terms in (1) interrelate as the properties of the crystal change. For example, as atoms are added to random locations in the unit cell (keeping *V*_cell_ fixed for the moment) the structure factor of the unit cell (*F*) increases as the square root of the number of atoms in the unit cell (*N*_cell_) and hence the intensity of a fully recorded spot (*I*, in photons) is proportional to *N*_cell_. Conversely, if *V*_cell_ is increased while keeping *V*_xtal_ and the total number of atoms in the crystal constant, then the number of unit cells (*V*_xtal_/*V*_cell_) decreases while *N*_cell_ increases. This causes *F* to increase as the square root of *V*_cell_, so *F* ^2^ cancels one *V*_cell_ term and the net effect of reorganizing a fixed number of atoms into larger cells is that individual spot intensities decrease proportionally to *V*_cell_. Since the number of relps in a given volume of reciprocal space is also proportional to *V*_cell_, the total summed intensity of all spots does not change and remains proportional to the number of atoms in the X-ray beam regardless of how these atoms are divided into unit cells. Another way to reach the same conclusion is by the simple fact of conservation of scattered photons: a given number of atoms will scatter a fixed number of photons and this number is dictated by the elastic scattering cross-section of these atoms. The arrangement of the atoms affects the direction in which these photons are scattered but cannot change their number and in the limiting case of very small unit cells that have no relps intersecting the Ewald sphere all of these photons are scattered in the forward direction (the relp with index *hkl* = 000).

The number of scattering atoms per unit volume in protein crystals varies with solvent content because the atoms of dis­ordered solvent contribute only very weakly to high-angle Bragg peaks (Tronrud, 1997 ▶; Afonine *et al.*, 2005 ▶). Therefore, the number of atoms contributing to spots at a given resolution beyond ∼4 Å can be taken as the number of ordered (protein) atoms in the unit cell,
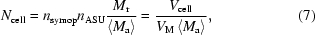
where *N*_cell_ is the total number of ordered atoms in the unit cell (including hydrogen), *n*_symop_ is the number of symmetry operators in the space group, *n*_ASU_ is the number of protein molecules in the asymmetric unit, *M*_r_ is the molecular weight of the protein (Da or g mol^−1^), 〈*M*_a_〉 is the number-averaged protein-atom mass (*M*_r_/*N*_protein_ ≃ 7.13 g mol^−1^), *N*_protein_ is the total number of ordered atoms in the protein (including hydrogen), *V*_cell_ is the volume of the unit cell (in Å^3^) and *V*_M_ is the Matthews coefficient (Å^3^ Da^−1^; Matthews, 1968 ▶). Since protein consists of more than one kind of atom, the effective per-atom structure factor *f*_a_ is given by the number-weighted average of the square structure factors of each atom type,
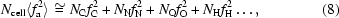
where 〈*f*_a_^2^〉 is the number-averaged squared atomic structure factor of protein (electron^2^), *N*_Ee_ is the number of ordered atoms of element Ee and *f*_Ee_ is the atomic structure factor of element Ee (electron equivalents). In this work, atomic form factors were calculated using the five-Gaussian fit approximation used by the *CCP*4 suite (Collaborative Computational Project, Number 4, 1994 ▶; Winn, 2003 ▶) and tabulated in *International Tables for Crystallography* Vol. *C* (Maslen *et al.*, 1999*b* ▶). Given the atomic composition of protein provided in §[Sec sec2.5]2.5, this average atomic structure factor of protein is roughly equivalent to that of boron (*f*_a_ ≃ 5 electrons for forward scattering). This is because half of the atoms in protein are hydrogen and this brings down the number-averaged quantities 〈*f*_a_^2^〉 and 〈*M*_a_〉. However, the quotient *f*_N_^2^/14 is at worst 14% greater than 〈*f*_a_^2^〉/〈*M*_a_〉 between 1.5 and 4 Å resolution, so if 14% error in calculated intensity is tolerable then protein can be considered to be made of an equal mass of nitrogen.

Note that (8) only applies for ∼4 Å resolution and better, where the approximations of Wilson (1942 ▶, 1949 ▶) hold, and recall that the structure factors *F* and *f*_a_ depend on the *d*-­spacing of the spot (*d*). The contribution of each atom is also modified by an atomic *B* factor (Maslen *et al.*, 1999*a* ▶) identical to those listed in the Protein Data Bank (PDB; Berman *et al.*, 2002 ▶). It is important to note that the *B* factor is the only model of intrinsic crystal disorder used in this work. Although there is reason to believe that disorder in crystals is more complicated than this (Welberry, 2004 ▶), *B* factors remain the formalism for describing disorder in crystallographic refinement (Tronrud, 2007 ▶; Brunger, 2007 ▶; Murshudov *et al.*, 1997 ▶, 1999 ▶; Winn *et al.*, 2003 ▶; Zwart *et al.*, 2008 ▶). Fundamentally, Debye’s argument (Debye, 1915 ▶) was that the effect of atomic displacements from their ideal lattice points is dominated by the mean square atomic displacement 〈*u*_*x*_^2^〉, a result that Waller (1923 ▶, 1925 ▶) related to temperature and Ott (1935 ▶) derived rigorously (James, 1962 ▶). *B* factors form a resolution-dependent ‘weight’ for the contribution of each atom and atoms with low *B* factors will contribute a larger fraction of the total scattering at high angles than atoms with high *B* factors. However, as long as the contribution of each protein atom is similar at a given resolution of interest we may substitute the Wilson *B* factor (Wilson, 1949 ▶; Shmueli & Wilson, 1999 ▶) for all the atomic *B* factors and arrive at a general expression for the average square structure factor of a unit cell,
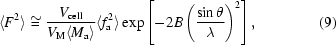
where 〈*F* ^2^〉 is the average value of the squared structure factor of the unit cell (electrons^2^), *V*_cell_ is the volume of the unit cell (Å^3^), *V*_M_ is the Matthews coefficient (Å^3^ Da^−1^ or Å^3^ mol g^−1^; Matthews, 1968 ▶), 〈*M*_a_〉 is the number-averaged protein-atom mass (*M*_r_/*N*_protein_ ≃ 7.1 g mol^−1^), 〈*f*_a_^2^〉 is the number-averaged squared atomic structure factor of protein (electrons^2^), *B* is the average (Wilson) *B* factor (Å^2^), θ is the the Bragg angle and λ is the X-ray wavelength (Å).

Since 〈*f*_a_〉 and 〈*M*_a_〉 are essentially constants for protein and *V*_M_ also has a restricted range (Matthews, 1968 ▶; Kantardjieff & Rupp, 2003 ▶), it is readily apparent that substituting 〈*F* ^2^〉 from (9) for |*F*|^2^ in (1) does indeed cancel one of the 1/*V*_cell_ terms. For example, if *V*_M_ = 2.5 Å^3^ Da^−1^, *d* = 2.5 Å and *B* = 0, (9) reduces to 〈*F* ^2^〉 ≃ 0.2*V*_cell_. That is, given two protein crystals with the same *V*_xtal_ (and Wilson *B* factor) but one with *V*_cell_ twice that of the other, the average spot intensity from the large unit-cell crystal will be half of that from the smaller unit-cell crystal.

### 2.8. Exposure time and multiplicity

The exposure time (*t*) does not appear explicitly in (1) because it is hidden in the rotation speed ω = ΔΦ/*t*, where ΔΦ is the rotation covered during an exposure (in radians). What happens if the crystal is not rotated during the exposure? Does the spot intensity become infinite? Of course not, but in reality it does approach the intensity of the incident beam as the mosaic spread approaches zero, the mosaic domain volume becomes large and the X-ray beam becomes perfectly monochromatic and parallel. This limiting case is routinely achieved with the perfect silicon crystals used in monochromators, where nearly 100% of X-rays at a desired wavelength are reflected, a treatment which requires the dynamical theory of diffraction (Authier, 2004 ▶). (1) is based on what is known as the kinematical approximation to the dynamical theory and assumes that the mosaic domains are small compared with the attenuation length of the X-rays in the crystal and that the drop in the main-beam intensity owing to diffraction is negligible, which is generally a very good assumption for protein crystals (see μ^−1^ values at the end of §[Sec sec2.5]2.5).

What value then should we choose for ΔΦ? It cannot be smaller than the mosaic spread if we are to fully record a spot, but since we are interested in collecting a complete data set we must set ΔΦ to the full rotation range of the data set and set *t* to the total accumulated exposure time of the data set (*t*_DS_). The average angular velocity for recording each spot is then simply ω = ΔΦ/*t*_DS_. Now, several spots belonging to the same unique *hkl* index may be observed in a given data set, so account must be taken of the extra signal available from merging equivalent observations. Any relp that is not in the blind region (see §[Sec sec2.3]2.3) will cross the Ewald sphere twice during a 360° rotation, as will the Friedel mate. Therefore, a crystal belonging to a space group with *n*_symop_ symmetry operators will produce a total of 4*n*_symop_ observations of each accessible unique *hkl* index (merging Friedel mates). For simplicity, we will use 360° for ΔΦ and multiply the single-spot intensity by 4*n*_symop_,
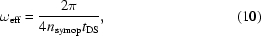
where ω_eff_ is the effective angular velocity for the data set (radians s^−1^), 2*π* = 360°, *n*_symop_ is the number of symmetry operators in the space group and *t*_DS_ is the total accumulated exposure time of a complete data set (s). That is, ω_eff_ is the angular velocity of a 360° data set. In practice, a data-collection strategy (Dauter, 1999 ▶) is often devised to take advantage of reciprocal-space symmetry and collect a complete data set with ΔΦ < 360°, but such strategies are generally planned to finish at the end of the crystal’s useful life (discussed in Appendix *C*) so *t*_DS_ is the same. The per-image exposure time is increased and this decreases ω, but it also decreases the number of observations, so ω_eff_ formally does not change. That is, a strategized data set will contain fewer but proportionally brighter spots and the radiation-damage-limited photon count is independent of the collection strategy.

This does not mean a data-collection strategy is useless! A well designed strategy minimizes the noise accumulation and resource consumption inherent in using a given set of equipment, such as the read-out noise of a CCD chip or the time required to collect the data, but a discussion of these concerns is beyond the scope of this work. Here we are interested in the absolute minimum crystal size, even given an ideal diffracto­meter, so we assume that the only source of noise in a spot is the photon-counting noise (shot noise) of the Bragg-scattered photons themselves and all other sources of noise, including the contribution of background scattering, are assumed to be negligible.

### 2.9. Absorption and dose

The attenuation factor *A* described in §[Sec sec2.5]2.5 is often incorrectly referred to as an ‘absorption factor’, but attenuation refers to every process for removing photons from a beam of light, including scattering. Absorption is the process of transferring energy from the beam into the substance of the crystal and the amount of energy ‘deposited’ into a sample per unit mass is the dose (Gy or J kg^−1^). The mass of our spherical crystal is simply its density (ρ) multiplied by its volume *V*_xtal_ = 4π*R* ^3^/3 and the available energy is the photon energy (*E*_ph_) multiplied by the number of photons that were not transmitted. The latter is the number of incident photons (*I*_beam_ × π*R* ^2^) multiplied by the fraction 1 − *T*_sphere_(0, μ, *R*) (see equation 5). In this way, the calculation of dose is related to that of the attenuation factor (*A*) because the process of dose deposition begins with a photon–atom interaction, but not every interaction deposits the full photon energy as dose. Some photons are merely scattered, depositing little or no energy, and in some cases absorbed energy is fluoresced away (Paithankar *et al.*, 2009 ▶). Seltzer (1993 ▶) accounted for such energy-loss mechanisms by assuming that only low-energy charged particles represent a ‘deposit’ of dose and tabulated the result as the mass energy-absorption coefficient μ_en_. Operationally, calculating absorption instead of attenuation amounts to substituting μ_en_ for μ_xtal_ in (5), which leads to
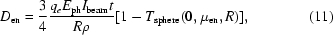
where *D*_en_ is the dose in Gy (J kg^−1^), *q*_e_ is the electron charge (1.6022 × 10^−19^ J eV^−1^), *E*_ph_ is the photon energy (eV/photon), *I*_beam_ is the incident-beam intensity (photons s^−1^ m^−2^), *t* is the exposure time (s), ρ is the density of the sphere material (kg m^−3^ or g l^−1^), *R* is the radius of the sphere (m) and μ_en_ is the mass energy-absorption coefficient of the sphere material (m^−1^). The subscript ‘en’ denotes the use of the Seltzer (1993 ▶) coefficient. Note that the 1/*R* term in (11) is effectively can­celled by the *T*_sphere_ term for typical wavelengths and crystal sizes. Take, for example, a cube-shaped crystal of the same width as our sphere, which will transmit *T*_cube_ = exp(−μ·2*R*), and since the limit of 1 − exp(−*x*) as *x* → 0 is *x*, one can see that the (1 − *T*) term approaches μ·2*R* when most of the beam is transmitted. This is generally the case for protein crystals, but we will keep (11) in its exact form and continue to use the spherical crystal model for dose and attenuation to avoid complicating our analysis of the attenuation factor (*A*) against resolution with the corners of a rotating cube-shaped crystal.

If the beam profile is not flat (the constant *I*_beam_ case assumed here and in §[Sec sec2.2]2.2) then some parts of the crystal will absorb more dose than others and these high-dose regions will ‘count’ more in the diffraction pattern than the low-dose regions because they experience a brighter part of the beam (see equation 1). Formally, we may deal with non-uniform beams as discussed in §[Sec sec2.2]2.2 by breaking up the crystal into tiny cubes that do experience a constant *I*_beam_ and then summing the resulting diffraction patterns [using equation (4) to account for the attenuation of each incident and diffracted beam]. However, we shall see in §[Sec sec2.11]2.11 and Appendix *C* that such a treatment is unnecessary because the damage-limited photon yield per spot is independent of *I*_beam_, obviating the need to integrate over the beam profile. That is, given a long enough exposure time every part of the crystal will eventually ‘burn out’ and contribute whatever it will contribute to the diffraction pattern. Therefore, for simplicity, we keep the ‘average dose’ given by (11) and assume that the entire crystal is ‘evenly cooked’ with no significant microscopic variation in the dose across the crystal.

### 2.10. Photoelectron escape and the meaning of ‘dose’

Cowan, Nave and Hill (Nave & Hill, 2005 ▶; Cowan & Nave, 2008 ▶) have pointed out that as the size of a protein crystal (*R*) is reduced it eventually approaches the size of a primary photoelectron track (*R*_PE_) and the electrons themselves will start to escape. When this happens, the energy ‘deposited’ within the crystal (dose) will be less than that predicted by (11).

In general, dose calculations are not simple and although a sphere is the simplest possible shape, (11) comes with certain caveats. For example, if *R* becomes large compared with μ_en_^−1^ of the crystal material then some fraction of the photons scattered from the core will be absorbed before escaping the sphere and some of the energy discounted to scattering by Seltzer must be added back to the dose. A similar correction must also be made for energy assumed to be lost to fluorescence if *R* becomes large compared with μ_en_^−1^ for the energy of the fluorescent photons (Paithankar *et al.*, 2009 ▶). Conversely, as *R* becomes comparable to *R*_PE_ the dose given by using μ_en_ will be too high.

Fundamentally, the flow of energy between attenuation and radiation damage is a shower of particles which quickly divides the energy of the initial photon among a large number of atoms distributed in space. For example, a photoelectric absorption event results in an excited atom and a photoelectron (Einstein, 1905 ▶; Hubbell, 2006 ▶) and the excited atom then relaxes by emitting a fluorescent photon (Moseley, 1913 ▶) or more electrons *via* Auger (Meitner, 1922 ▶; Auger, 1925 ▶) or Coster–Kronig (Coster & Kronig, 1935 ▶) processes (ICRU, 1983 ▶). These particles travel some distance before colliding with another atom and this cascade continues, with the number of excited atoms increasing and the magnitude of transferred energy decreasing with each subsequent collision. However, the distribution of events is not entirely random, as energy transfer requires an allowed electronic transition in the material. Initially, at high energies, the number of allowed transitions is small (photoelectric absorption by deep shells and scattering), but the list of possible transitions increases dramatically at lower energy. Chemical transformations take place once the magnitude of energy transfer approaches that of the strongest chemical bonds in the sample (∼1 eV or 100 kJ mol^−1^) and there are a very large number of such states excited by a single X-ray photon.

Unfortunately, such a complete treatment of energy flow is not only beyond the scope of this work but is beyond the current understanding of radiation physics in complex sub­stances. For example, the available transitions or ‘oscillator strength’ in pure water between 30 and 100 eV are still poorly understood (Garrett *et al.*, 2004 ▶). Dose calculations with particle-tracking simulation codes such as *EGS* (Nelson *et al.*, 1985 ▶; Kawrakow & Rogers, 2001 ▶; Edimo *et al.*, 2008 ▶) or *MCNP* (Hendricks *et al.*, 2000 ▶; Chiavassa *et al.*, 2005 ▶; Chibani & Li, 2002 ▶) take into account carefully tabulated single- and double-differential cross-sections of all known interactions between atoms, photons and electrons, but once a particle energy drops below 1 keV it is added to the ‘dose’ because this is where most of the cross-section tabulations end. This means that even these highly sophisticated dose calculations will systematically underestimate track lengths by the range of 1 keV electrons. Cole (1969 ▶) measured this to be ∼0.06 µm in collodion plastic, so *MCNP* will overestimate the dose to crystals of the order of 60 nm and smaller.

Perhaps the most important caveat is that photoelectron escape formally violates the fundamental dosimetric principle of charged-particle equilibrium (CPE; Attix, 1986 ▶; Moussa *et al.*, 2006 ▶), making simulation results difficult to interpret. The concern over violating CPE arises because more than half of the energy ‘deposited’ by a photoelectron is not in the form of ionizations but rather charge-neutral electronic excitations. Significantly more energy is deposited in this non-ionizing form at the beginning of an electron track than at the end (ICRU, 1983 ▶). No doubt this energy destabilizes the molecules that receive it, but probably not in the same way as energy deposited by ionizing interactions. Since it is not clear which kind of energy transfer is relevant to the fading of diffraction spots, the impact of ‘dose’ may vary along the track.

To date, all dose-calibrated radiation-damage measurements have been conducted with samples larger than the relevant photoelectron tracks and the dose has been calculated using coefficients such as μ_en_, so we shall continue to use μ_en_ for dose in this work. However, in anticipation of future developments we shall introduce a Nave–Hill ‘capture fraction’ f_NH_ to represent the fraction of the conventionally calculated dose *D*_en_ from (11) remaining in the crystal and contributing to the ‘true’ dose (*D*_reso_) that is relevant to resolution-degrading chemical transformations. For large crystals in ∼1 Å X-ray beams we assert that f_NH_ = 1 and in our highly symmetric case of a uniform beam and a spherical crystal in a vacuum this correction can only depend on the radius of the crystal *R* and the X-ray photon energy (*E*_ph_). Although an exact expression cannot be derived at this time, a rough estimate of f_NH_ is useful for detecting when a crystal has reached a size where the Nave–Hill effect may have a significant impact. Since photoelectrons are preferentially emitted in a direction normal to the incident beam and deposit energy more-or-less evenly along their track, it is assumed here that the rough effect of photoelectron escape will be to enlarge the volume over which the dose is deposited in a single direction and thereby reduce the dose to the crystal by a fraction
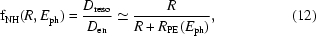
where *E*_ph_ is the photon energy (eV/photon), *R* is the radius of the spherical crystal (m) and *R*_PE_(*E*) is the range of a photoelectron of energy *E* derived by Cole (1969 ▶) (m). Note that for simplicity the *K*-shell energy of the atom that emits the photoelectron has not been deducted from the photon energy before applying it to Cole’s formula, nor have Compton electrons been considered, but these are not likely to be the largest source of error in (12). It must be stressed that this equation is a very rough estimate only and could easily be off by a factor of two or more when *R* << *R*_PE_. However, it is instructive to show that f_NH_ is expected to reduce the dose roughly as the first power of *R* once *R* becomes less than *R*_PE_.

**Figure 2:**
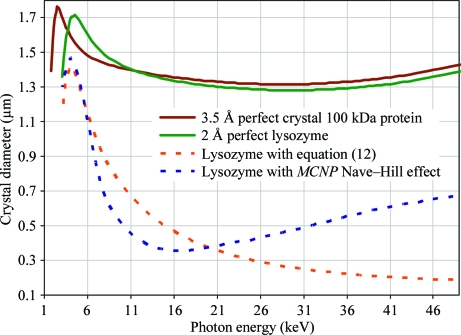
Wavelength-dependence of the minimum required crystal size. All plotted calculations used *V*_M_ = 2.4 Å^3^ Da^−1^, Wilson *B* = 0 and four photons/*hkl* in the indicated resolution bin. The crystal size required for 2 Å data from lysozyme and 3.5 Å data from a 100 kDa protein are essentially identical as these cases balance scattering power with data-quality requirements. Solid lines were calculated neglecting photoelectron escape (f_NH_ = 1) and dotted lines represent two different models for photoelectron loss: that given by (12) (orange) and a full particle-tracking dose calculation with the program *MCNP* (blue). The sharp reversal of the curves at low energy is a consequence of the onset of backscattering, where the Lorentz factor spikes.

To demonstrate the potential variability of f_NH_ calculations, we conducted *MCNP* (Hendricks *et al.*, 2000 ▶) simulations of a sphere with radius *R* and the density and atomic composition of a protein crystal given in §[Sec sec2.5]2.5 illuminated in a vacuum by X-­rays of various energies. The resulting minimum crystal sizes are plotted against those obtained using (12) in Fig. 2 ▶. Note that certain conclusions such as the optimum photon energy to use clearly depend on how f_NH_ is calculated. The *MCNP* calculation is probably more reliable than the simplistic model in (12), but the caveats mentioned above have yet to be addressed.

### 2.11. Radiation damage

**Figure 3:**
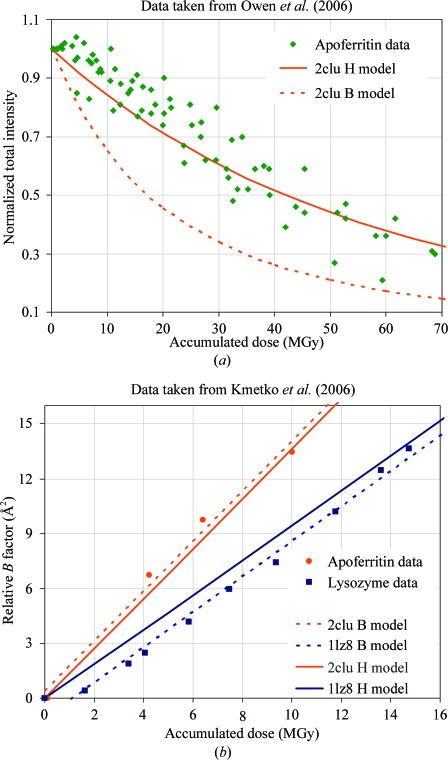
Radiation-damage model. The observations made by Owen *et al.* (2006 ▶) and Kmetko *et al.* (2006 ▶) are reproduced with permission from the original publishers and plotted against predicted curves derived from two alternative radiation-damage models. The ‘H model’ is an exponential decay of spot intensity with dose and the ‘B model’ is the dose-dependent *B*-factor model suggested by Kmetko *et al.* (2006 ▶). The ‘H model’ pre­dictions were made by applying (13) to intensities derived from the observed structure-factor file deposited with the indicated PDB entry and then computing the sum of all intensities (*a*) followed by scaling the ‘simulated damage’ intensities to the ‘zero-dose’ intensities (*b*) using the procedure described by Kmetko *et al.* (2006 ▶). The ‘B model’ prediction curves (dotted lines) were prepared similarly except that the ‘simulated damage’ intensities were generated by applying the relevant dose-dependent *B* factor reported by Kmetko *et al.* (2006 ▶). All ‘H model’ curves (solid lines) used the same value of *H* (10 MGy Å^−1^) and therefore may explain the dissimilar ‘sensitivity parameter’ observed by Kmetko *et al.* (2006 ▶) for apoferritin and lysozyme (orange circles *versus* blue squares, respectively). It is clear from (*a*) that the ‘B model’ is at odds with the observations of Owen *et al.* (2006 ▶) (green diamonds), although the same predicted intensities are in very good agreement with the data points from Kmetko *et al.* (2006 ▶) (orange circles). Agreement between these two studies is restored, however, if we accept the ‘H model’ where the resolution-dependence of radiation damage is exponential as opposed to a Gaussian (B model).

The radiochemical mechanism behind the fading of diffraction spots is not presently clear (Garman & Nave, 2009 ▶), but the connection to dose has been calibrated experimentally. Specifically, it was pointed out by Holton (2009 ▶) and Howells *et al.* (2009 ▶) that the general trend reported by Howells *et al.* (2009 ▶), namely *D*_1/2_ ≃ 10*d* MGy, where *d* is the feature size in Å, is remarkably consistent with the independent observations of both Owen *et al.* (2006 ▶) and Kmetko *et al.* (2006 ▶) (see Fig. 3 ▶) if the average spot intensity at a given resolution fades exponentially,
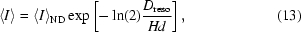
where 〈*I*〉 is the average spot intensity (photons/spot) after absorbing a dose *D*_reso_, 〈*I*〉_ND_ is the average spot intensity (photons/spot) expected in the absence of radiation damage, ln(2) is the natural log of two (∼0.7), *D*_reso_ is the deposited dose that is relevant to spot fading (MGy), *H* is the criterion of Howells *et al.* (2009 ▶) (10 MGy Å^−1^) and *d* is the *d*-­spacing in Å.

Note that here we use *D*_reso_ because it was defined in the last section as the resolution-degrading dose, but for currently available spot-fading data this is the same as *D*_en_ from (11) (f_NH_ = 1). We use angle brackets 〈〉 to emphasize that (13) describes the decay of average spot intensity at a given *d*-­spacing, as opposed to the decay of any particular spot. Realistically, individual spots may follow different paths of decay that are not necessarily exponential (Blake & Phillips, 1962 ▶; Banumathi *et al.*, 2004 ▶), but in this work we are only interested in the average spot intensity in a given resolution bin and the argument for (13) is based largely upon spot-fading measurements.

The meta-analysis of Howells *et al.* (2009 ▶) did not include the observations made by Owen *et al.* (2006 ▶) or Kmetko *et al.* (2006 ▶), but we reproduce in Fig. 3 ▶ the observations presented in these works superimposed on predictions made by our radiation-damage model (H model) and the dose-dependent *B*-factor model (B model) suggested by Kmetko *et al.* (2006 ▶). We selected PDB entries 2clu and 1lz8 as representative of apoferritin and lysozyme, respectively, because 2clu claims a similar resolution limit to that observed in Owen *et al.* (2006 ▶) and 1lz8 is the entry for lysozyme reported by Kmetko *et al.* (2006 ▶). It should be noted that the same value of *H* (10 MGy Å^−1^) was used for all ‘H model’ curves in Fig. 3 ▶ and this was not ‘fitted’ to the plotted data points in any way, so the agreement between all observations and the ‘H model’ predictions (solid lines) is quite remarkable. In fact, the ‘H model’ predictions in Fig. 3 ▶(*b*) were intentionally offset to pass through the origin so that the ‘H model’ lines would not obscure the least-squares fitted lines of the ‘B model’. In this work we use the ‘H model’ because it is in best agreement with both these studies as well as 20 other radiation-damage experiments surveyed by Howells *et al.* (2009 ▶).

However, spot-fading experiments measure the same spots over and over again and we are interested in the total accumulated intensity 〈*I*〉_DL_ at the ‘damage limit’ (*T*_DL_), so we must integrate (13) over time. This integral is performed in Appendix *C*, where we show that integrating over an exponential decay is equivalent to accumulating a nondecaying intensity for less time, and applying the proportionality constant gives
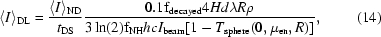
where 〈*I*〉_DL_ is the average damage-limited intensity (photons/spot) at a given resolution, 〈*I*〉_ND_ is the average spot intensity (photons/spot) expected in the absence of radiation damage, *t*_DS_ is the exposure time for the data set (s), 0.1 is a factor for converting three units λ from Å to m, ρ from g cm^−3^ to kg m^−3^ and MGy to Gy, f_decayed_ is the fractional progress toward completely faded spots at end of the data set, *H* is Howells’s criterion (10 MGy Å^−1^), *d* is the resolution of interest (Å), λ is the X-ray wavelength (Å), *R* is the radius of the spherical crystal (m), ρ is the density of the crystal (∼1.2 g cm^−3^), f_NH_ is the Nave–Hill dose-capture fraction, *h* is Planck’s constant (6.626 × 10^−34^ J s), *c* is the speed of light (299 792 458 m s^−1^), *I*_beam_ is the incident-beam intensity (photons s^−1^ m^−2^) and μ_en_ is the mass energy-absorption coefficient of the sphere material (m^−1^). Note that the ‘damage limit’ was defined in Appendix *C* as the point when spot intensity has decayed by some fraction (f_decayed_) of the initial ‘undamaged’ value. For example, Owen *et al.* (2006 ▶) recommended ending the data collection when the average spot intensity fades to ∼0.7 of the undamaged value (f_decayed_ = 0.3), but the level of concern over radiation damage for a particular project may inspire some investigators to exceed this limit or set a more conservative limit (Holton, 2009 ▶).

The value of 〈*I*〉_ND_ is simply the average value of spot intensity as given by (1) and computation of this average was accomplished by replacing the terms in (1) that vary from spot to spot with their average values and also by substituting ω_eff_ from (10) to convert spot intensities into merged *hkl* intensities,
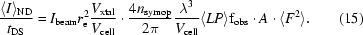
We may now substitute 〈*I*〉_ND_/*t*_DS_ from (15) into (14) and then replace 〈*LP*〉f_obs_, 〈*F* ^2^〉, *V*_cell_ and *V*_xtal_ with their expanded forms from (6), (9), (7) and 4π*R* ^3^/3, respectively, to yield the fully qualified expression for damage-limited spot intensity,
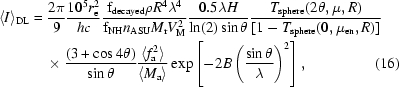
where 〈*I*〉_DL_ is the average damage-limited intensity (photons/*hkl*) at a given resolution, 10^5^ is a factor for converting four units: *R* from µm to m, *r*_e_ from m to Å, ρ from g cm^−3^ to kg m^−3^ and MGy to Gy, *r*_e_ is the classical electron radius (2.818 × 10^−15^ m), *h* is Planck’s constant (6.626 × 10^−34^ J s), *c* is the speed of light (299 792 458 m s^−1^), f_decayed_ is the fractional progress toward completely faded spots at the end of the data set, ρ is the density of the crystal (∼1.2 g cm^−3^), *R* is the radius of the spherical crystal (µm), λ is the X-ray wavelength (Å), f_NH_ is the Nave–Hill dose-capture fraction (1 for large crystals; Nave & Hill, 2005 ▶), *n*_ASU_ is the number of proteins in the asymmetric unit, *M*_r_ is the molecular weight of the protein (Da or g mol^−1^), *V*_M_ is the Matthews coefficient (∼2.4 Å^3^ Da^−1^), *H* is Howells’s criterion (10 MGy Å^−1^), θ is the Bragg angle, 〈*f* ^2^_a_〉 is the number-averaged squared structure factor per protein atom (electron^2^), 〈*M*_a_〉 is the number-averaged atomic weight of a protein atom (∼7.1 Da), *B* is the average (Wilson) temperature factor (Å^2^), μ is the attenuation coefficient of the sphere material (m^−1^) and μ_en_ is the mass energy-absorption coefficient of the sphere material (m^−1^). Note that the incident-beam intensity (*I*_beam_) is missing from this equation because spot intensity was integrated out to the ‘damage limit’ where the average spot has decayed by a given fraction (f_decayed_). Note that the crystal symmetry is also missing, as the *n*_symop_ term from (10) was cancelled by another *n*_symop_ term in the expression for the average structure factor (7), implying that the damage limit is more closely related to the number of molecules in the crystal than it is to the number of unit cells. One *R* in the *R* ^4^ term is effectively cancelled by the (1 − *T*) term for all but the very largest protein crystals and one λ term is roughly cancelled (within ∼30% between 7 and 17 keV) by the 〈*LP*〉f_obs_ factor.

Although (16) may appear somewhat intimidating, it is both instructive and useful to examine it in this expanded form as this eases the incorporation of different macromolecule types, radiation-damage models and crystal shapes. For example, 〈*f*_a_^2^〉, 〈*M*_a_〉, μ and μ_en_ may be replaced with appropriate values for nucleic acids. The ln(2) term arises from the definition of *H* as the dose required to reduce spot intensities at a given *d*-­spacing (*d* = 0.5λ/sinθ) by half, so *Hd* and ln(2) are grouped together. Crystals that are more sensitive than normal to radiation damage per unit of dose, as was reported for dodecin by Murray *et al.* (2005 ▶), may be represented by using a smaller value of *H* and a more sophisticated resolution-dependent damage model might replace *Hd*/ln(2) with an arbitrary function *H*(*d*). Also, considering the crystal to be a cube with edge 2*R* instead of a sphere of radius *R* simply changes the leading 2π/9 term to unity and replaces *T*_sphere_ with exp(−μ_en_2*R*). The increased scattering power of the cube arises because (2*R*)^3^ is roughly twice 4π*R* ^3^/3 and the damage-limited intensity (photons/*hkl*) scales linearly with crystal volume.

## 3. Results and discussion

We are now prepared to calculate the diameter of the smallest protein crystal that can be expected to produce a complete data set on an ideal diffractometer: a very large perfect detector, a perfect shutter and a perfect spindle with a uniform and flicker-free X-ray beam bathing a spherical protein crystal in a vacuum. The noise from such a machine is dominated by photon counting, so if we require a signal-to-noise ratio (SNR) of 2.0 in the outer resolution bin of say 2 Å then the average *hkl* in this bin must accumulate at least four photons (*I*/σ = *I*/*I* ^1/2^). If there are other sources of noise, such as background scattering, then more than four photons will be required, but since it is theoretically possible to reduce background to a negligible level (see §[Sec sec3.2]3.2), we will begin with this limiting case.

### 3.1. Zero-background case

We begin by neglecting the Nave–Hill effect because it has yet to be measured and represents the greatest unknown in the dose calculation. With f_NH_ = 1, (16) predicts that a 1.2 µm diameter sphere of perfect lysozyme crystal (*B* = 0; *M*_r_ = 14 300 Da; *V*_M_ = 2.0 Å^3^ Da^−1^) in a beam of 1 Å X-rays will scatter an average of 4 photons/*hkl* (〈*I*〉_DL_) at 2 Å resolution before the radiation-damage limit is reached (f_decayed_ = 0.3). This limit is independent of exposure time or beam flux since the total accumulated fluence (photons/area) is dictated by the damage limit.

If we now involve f_NH_ from (12) or from *MCNP* simulations then the four-photon lysozyme crystal size shrinks to 0.5 or 0.34 µm, respectively. In addition to this, if we allow the spots to fade away completely (f_decayed_ = 1) then 0.81 µm (f_NH_ = 1), 0.28 µm (equation 12) or 0.19 µm (MCNP) crystals will yield 4 photons/*hkl* at 2 Å. There are a number of reasons why complete decay is not a realistic damage limit, not the least of which is the biological relevance of the results (Owen *et al.*, 2006 ▶), but it is instructive to consider an infinite exposure time here because photon counting is the only kind of noise that is theoretically impossible to eliminate.

Immediately, the next questions to ask are how this limit is influenced by the choice of photon energy, desired resolution, degree of disorder in the crystal and molecular weight of the protein or combinations thereof. (16) is the exact formula for relating all these quantities, but as the questions to be asked occupy a large multidimensional parameter space it is in­structive to graph the influence of each parameter separately. Since many of the variables in (16) change with the X-­ray wavelength, we begin by plotting the minimum crystal size against photon energy in Fig. 2 ▶. This graph is similar to the ‘*I*_E_’ quantity obtained by Arndt (1984 ▶), except that here the *y* axis is on an absolute scale. The energy-dependence is remarkably flat and this result is consistent with experimental observation (Gonzalez *et al.*, 1994 ▶). The ‘spike’ in crystal size at very low photon energy arises from a sharp upswing in 〈*LP*〉 when the relp grazes the back of the Ewald sphere just before f_obs_ drops to zero and the 2 Å curves stop at 3.1 keV because it is not possible to collect 2 Å data with wavelengths longer than 4 Å. The minimum-size curve for 4 photons/*hkl* at 3.5 Å from a perfect crystal of a 100 kDa protein is provided to fill this low-energy gap as well as demonstrate how simultaneously decreasing the scattering power and lowering the desired data quality can ‘coincidentally’ result in the same crystal size requirement.

**Figure 4:**
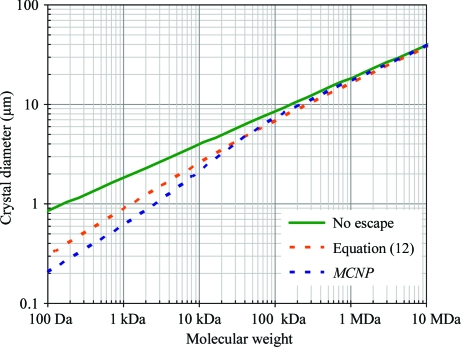
Molecular-weight dependence of the minimum required crystal size. All plotted calculations used *V*_M_ = 2.4 Å^3^ Da^−1^, 1 Å radiation, 2 Å spots and *B* = 24 Å^2^. Without photoelectron escape, the required crystal volume is simply proportional to molecular weight and the two different models of photoelectron escape considered here are shown to have significant yet different effects for crystals smaller than a few micrometres wide, as this is the linear dimension of a photoelectron track (*R*_PE_).

Graphs of minimum crystal size against molecular weight (Fig. 4 ▶), *n*_ASU_, f_decayed_, *H* and absorption coefficients are all very similar because each of these terms scales linearly with crystal volume. An examination of (16) reveals that these variables are not strongly coupled to any others if *R* << μ^−1^, as absorption is proportional to *R* and attenuation is negligible in this case. The solvent content *V*_M_ dependence is also not graphed because this is just a plot of a square-root function passing through 1.2 µm for *V*_M_ = 2.0 Å^3^ Da^−1^, λ = 1 Å, *d* = 2 Å and *B* = 0.

**Figure 5:**
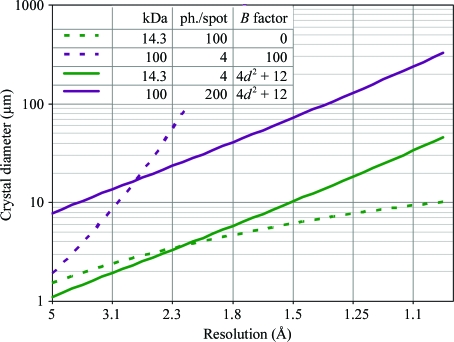
Resolution-dependence of the minimum required crystal size. All plotted calculations used *V*_M_ = 2.4 Å^3^ Da^−1^ and 1 Å radiation. The Wilson *B* factor strongly affects the curvature of the plot of the required crystal size for a given number of photons, but applying the empirical formula shown serendipitously simplifies this analysis, as described in the text.

The graph of minimum crystal size against desired resolution may curve upward or downward depending on the value chosen for the Wilson *B* factor (dashed lines in Fig. 5 ▶) and indeed it is not surprising that the degree of disorder in a protein crystal has a strong influence on the diffraction limit. What is surprising is that if the *B* factor is always selected to follow the empirically derived formula (*B* = 4*d* ^2^ + 12) presented by Holton (2009 ▶), one obtains the straight solid lines in Fig. 5 ▶. This remarkable result appears to be a consequence of this *B*-factor formula effectively cancelling the resolution-dependence of the average atomic form factor (8), implying that the number of photons required to detect the weakest spots is relatively fixed from crystal to crystal. Regardless of the origin, Fig. 5 ▶ immediately suggests an empirical formula for the required crystal size given an observed resolution limit,
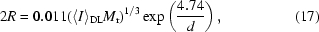
where 2*R* is the required diameter of the crystal (µm), 0.011 is a scale factor assuming *V*_M_ = 2.4 Å^3^ Da^−1^, 〈*I*〉_DL_ is the desired damage-limited intensity (photons/*hkl*) at a given resolution, *M*_r_ is the molecular weight of the protein (Da or g mol^−1^) and 4.74 = 4π^2^ *r*_a_^2^, where *r*_a_ is the radius of gyration of a protein atom (Å) and *d* is the resolution of interest (Å). This is not to say that a crystal of diameter 2*R* will diffract to resolution *d*, but rather that a crystal of a protein with mass *M*_r_ found to diffract to resolution *d* probably has a Wilson *B* factor that will require the crystal to be of diameter 2*R* to yield a complete data set. Until now, we have assumed that an outer resolution bin (〈*I*〉_DL_) need only gather 4 photons/*hkl*, but it appears that the ‘detection limit’ of current technology is much higher than this (described in the next section) and a value of 〈*I*〉_DL_ = 100–200 photons/*hkl* is suggested for the practical use of (17) depending on the background level.

### 3.2. Background scattering

X-ray background consists of scattering from air, aperture walls, fluorescence, disorder in the crystal and potentially many other sources. A full theoretical treatment of background and all other possible sources of noise in a diffraction experiment is well beyond the scope of this work, but we shall briefly describe here how the large gap between our calculated absolute minimum crystal size and those that have been determined experimentally is completely explained by background scattering alone.

A summary of experimental minimum crystal-size determinations was provided by Holton (2009 ▶), who related scattering power to data quality with an empirical ‘difficulty parameter’ (*n*_0_) that increases with the quality of data needed for ‘success’ and decreases as instrument capabilities improve. The ‘record’ for obtaining a complete data set was *n*_0_ = 3.1, but entering the parameters obtained in the last section into equation (3) of Holton (2009 ▶), *n*_xtals_ = 1 (number of crystals used), *d* = 2.0 Å (resolution limit), *B* = 0, *V*_M_ = 2.0 Å^3^ Da^−1^ and ℓ_*xyz*_ = 1.2 µm (crystal ‘size’), we obtain *n*_0_ = 0.2. This is a factor of 15 improvement over the ‘record’ and using ℓ_*xyz*_ = 0.34 µm, as expected from the more optimistic photoelectron escape model, we arrive at *n*_0_ = 0.0044, which is 700-fold less scattering power than has ever been used to collect a complete data set.

There are many possible reasons why extant beamlines may not have reached the theoretical limit, but what is clear is that more than four photons are presently required to detect the faintest spots. Indeed, the *n*_0_ = 3.1 case corresponds to 64 photons/*hkl* [if the cubic crystal volume in Holton (2009 ▶) is taken to be *V*_xtal_ here]. Formally, this must arise from additional noise inflating σ(*I*) beyond simply *I* ^1/2^, requiring increased *I* (photons/*hkl*) to bring *I*/σ(*I*) back up to 2.0. An obvious source of additional noise is background scattering, so we now generalize our formula for the average signal-to-noise ratio (SNR) in the outer resolution bin from simply 〈*I*〉^1/2^_DL_ to
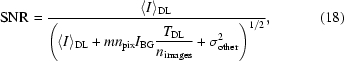
where 〈*I*〉_DL_ is the average damage-limited intensity (photons/*hkl*), *m* is the mean multiplicity (spots/*hkl*, counting partials as distinct spots), *n*_pix_ is the number of pixels involved in the average spot, *I*_BG_ is the average background scattering rate (photons pixel^−1^ s^−1^) at the resolution of interest, *T*_DL_ is the damage-limited exposure time of the data set (s), *n*_images_ is the number of diffraction images in the data set and σ_other_ is the root-mean-square of all other sources of noise (placed on a one-photon scale).

For a given camera and sample, the observed background photons/pixel on a single diffraction image will be proportional to the per-image exposure time (*t*_image_ = *T*_DL_/*n*_images_), indicating how *I*_BG_ is fixed for a given experiment. Since we are considering a damage-limited experiment, the total number of background photons that fall on the detector (*I*_BG_ *T*_DL_) is also fixed, regardless of how these photons are divided into images. The practice of ‘fine-slicing’ (Pflugrath, 1999 ▶) reduces *I*_BG_ *t*_image_, at the expense of increasing *m*, but in the limit of ‘infinite’ fine-slicing the quantity *mI*_BG_ *t*_image_ approaches a constant because the background that actually falls into the three-dimensional integration region of a given spot cannot be avoided by finer slicing. Very fine slicing will start to make other sources of noise important, such as detector read-out noise, so this and all other sources of noise are lumped into σ_other_ for completeness. Nevertheless, with our hypothetical ideal diffractometer σ_other_ will be negligible.

Choosing some reasonable parameters (*m* = 4, *n*_pix_ = 5 × 5) (18) is solved for SNR = 2.0 and 〈*I*〉_DL_ = 64 photons/*hkl* by *I*_BG_ *t*_image_ = 10 photons pixel^−1^. It must be stressed that this is a very rough approximation, particularly since *n*_0_ was not claimed to be accurate to better than a factor of two and such an error propagated through (18) becomes a factor of four in background level. Nevertheless, this *I*_BG_ *t*_image_ is exactly that observed near the faintest spots shown in Fig. 4 ▶ of Moukhametzianov *et al.* (2008 ▶), the source of our *n*_0_ = 3.1 ‘record’ (the detector registers 1.0 pixel levels per photon and has a ‘zero’ offset of 20 pixel levels).

The experience of the authors of this work is that 10 photons pixel^−1^ is on the low side of the range of background levels seen on typical diffraction images. It is more common to see hundreds of photons per pixel from crystals that only diffract to modest resolutions because the same disorder that leads to faint spots also produces diffuse scattering (James, 1962 ▶; Welberry, 2004 ▶). If we keep *n*_pix_ = 5 × 5 and *m* = 4 as above and *I*_BG_ *t*_image_ = 25, 100 or even 400 photons pixel^−1^, then satisfying SNR = 2 in (18) requires 〈*I*〉_DL_ to be 102, 202 or 402 photons/*hkl*, respectively.

Note that reducing the multiplicity (*m*) by collecting the bare minimum number of images will result in no net ‘gain’ so long as the damage limit is reached at the end of data collection because the increased exposure time per image will increase *I*_BG_ *t*_image_ to exactly compensate for any reduced multiplicity (*m*). On the other hand, considerable gains can be had by making the absolute background counts (photons pixel^−1^ s^−1^; *I*_BG_) lower, reducing the number of pixels occupied by spots on the detector (*n*_pix_) or both.

Background scattering can never be completely eliminated, but the noise it adds to the spots can be minimized by making the spot size very small. A detailed discussion of spot size is beyond the scope of this work, but theoretically very small spots can be achieved with a perfect protein crystal (no mosaic spread), a near-zero emittance beam of very short wavelength X-rays focused on an enormous and noiseless detector with no point-spread function, very small pixels and very fine rotation steps. Therefore, *I*_BG_ can be reduced to near zero, or at least to the point where the noise from background is insignificant (〈*I*〉_DL_ >> *mn*_pix_ *I*_BG_ *t*_image_ in equation 18), implying that (16) with 〈*I*〉_DL_ set to 4 photons/*hkl* represents an absolute and fundamental limit. That is, unless some way is found to change one of the parameters in (16), such as increasing *H* by mitigating the chemistry of global damage or decreasing f_NH_ with photoelectron escape, a lysozyme crystal smaller than 1.2 µm will never yield a complete data set to 2 Å.

### 3.3. Implications for micro-focus beams

The 1.2 µm size limit for perfect lysozyme crystals determined here does not imply that crystals and X-ray beams smaller than ∼1 µm are useless. If a complete data set cannot be obtained from one crystal then a multi-crystal strategy (Kendrew *et al.*, 1960 ▶; Dickerson *et al.*, 1961 ▶), a ‘needle-scanning’ strategy (Moukhametzianov *et al.*, 2008 ▶) or perhaps the ‘serial crystallography’ approach proposed by Starodub *et al.* (2008 ▶) may be employed, but the total scattering volume will have to add up to the volume of a sphere given by *R* in (16) using f_NH_ for the individual crystal size. For example, the volume needed for one crystal of a 100-crystal data set with final merged 〈*I*〉_DL_ = 4 photon/*hkl* is given by using 〈*I*〉_DL_ = 0.04 photon/*hkl* in (16).

Crystals with larger unit cells or more disorder (or both) will have to be larger than their ‘perfect lysozyme equivalent’ volume. For example, a lysozyme crystal with a more realistic Wilson *B* factor of 20 Å^2^ must be 2.8 µm wide to produce 4 photons/*hkl* in the 2 Å bin using the f_decayed_ = 0.3 damage limit and a 10 MDa asymmetric unit with *V*_M_ = 2.4 Å^3^ Da^−1^ and *B* = 61 Å^2^ must form a crystal 15 µm wide to produce 4 photons/*hkl* at 3.5 Å. However, as the present ‘detection limit’ appears to be of the order of 100 photons/*hkl* (*I*_BG_ *t*_image_ ≃ 100 photons pixel^−1^), these realistic lysozyme crystals will have to be 8.3 µm in diameter for 2 Å data, and 3.5 Å data from the 10 MDa case will require 43 µm crystals, limiting the usefulness of X-ray beams smaller than this.

## 4. Conclusions

The minimum useful protein crystal size is limited by the background photons that accumulate in the detector pixels occupied by a spot and current technologies seem to require of the order of 100 photons/*hkl* (after merging) to attain a signal-to-noise ratio of 2. The choice of X-ray wavelength appears to have only a minor impact on the damage-limited scattering power of a crystal, which remains proportional to the crystal volume and inversely proportional to both the molecular weight of the asymmetric unit and the square of the Matthews coefficient (Matthews, 1968 ▶) for all practical purposes. The resolution-dependence is complicated by the Wilson *B* factor, but relating *B* to *d*-spacing empirically revealed that damage-limited scattering power is proportional to exp(−14.2/*d*), where *d* is the *d*-spacing of interest. Dose reduction owing to photoelectron escape appears to be theoretically promising but difficult to predict and the current detection limit for spots will have to be overcome for this effect to be of practical use for typical single-crystal data sets at accessible photon energies.

## Supplementary Material

Supplementary material file. DOI: 10.1107/S0907444910007262/ba5148sup1.pdf

## Footnotes

1 Supplementary material has been deposited in the IUCr electronic archive (Reference: BA5148). Services for accessing this material are described at the back of the journal.

2 Note that there is also a ‘Lorentz factor’ in the Theory of Relativity, which has nothing to do with the Lorentz factor in crystallography other than sharing the same namesake.

## References

[bb1] Afonine, P. V., Grosse-Kunstleve, R. W. & Adams, P. D. (2005). *Acta Cryst.* D**61**, 850–855. 10.1107/S0907444905007894 PMC2808320 15983406

[bb2] Arndt, U. W. (1984). *J. Appl. Cryst.* **17**, 118–119.

[bb3] Arndt, U. W. & Wonacott, A. J. (1977). *The Rotation Method in Crystallography.* Amsterdam: North-Holland.

[bb4] Attix, F. H. (1986). *Introduction to Radiological Physics and Radiation Dosimetry.* New York: Wiley.

[bb5] Auger, P. (1925). *J. Phys. Radium*, **6**, 205–208.

[bb6] Authier, A. (2004). *Dynamical Theory of X-ray Diffraction*, revised ed. Oxford University Press.

[bb7] Azároff, L. V. (1955). *Acta Cryst.* **8**, 701–704.

[bb8] Banumathi, S., Zwart, P. H., Ramagopal, U. A., Dauter, M. & Dauter, Z. (2004). *Acta Cryst.* D**60**, 1085–1093. 10.1107/S0907444904007917 15159568

[bb9] Berger, M. J. & Hubbell, J. H. (1987). *XCOM: Photon Cross Sections on a Personal Computer.* National Bureau of Standards Internal Report NBSIR-87-3597. Gaithersburg: National Bureau of Standards.

[bb10] Berman, H. M. *et al.* (2002). *Acta Cryst.* D**58**, 899–907. 10.1107/s0907444902003451 12037327

[bb11] Blake, C. C. F. & Phillips, D. C. (1962). *Biological Effects of Ionizing Radiation at the Molecular Level*, pp. 183–191. Vienna: IAEA.

[bb12] Blundell, T. L. & Johnson, L. N. (1976). *Protein Crystallography.* New York: Academic Press.

[bb13] Bragg, W. L., James, R. W. & Bosanquet, C. H. (1921*a*). *Philos. Mag. Ser. 6*, **41**, 309–337.

[bb14] Bragg, W. L., James, R. W. & Bosanquet, C. H. (1921*b*). *Philos. Mag. Ser. 6*, **42**, 1–17.

[bb15] Bragg, W. L., James, R. W. & Bosanquet, C. H. (1922). *Philos. Mag. Ser. 6*, **44**, 433–449.

[bb16] Brunger, A. T. (2007). *Nature Protoc.* **2**, 2728–2733. 10.1038/nprot.2007.406 18007608

[bb17] Chiavassa, S., Lemosquet, A., Aubineau-Laniece, I., de Carlan, L., Clairand, I., Ferrer, L., Bardies, M., Franck, D. & Zankl, M. (2005). *Radiat. Prot. Dosimetry*, **116**, 631–635. 10.1093/rpd/nci063 16604715

[bb18] Chibani, O. & Li, X. A. (2002). *Med. Phys.* **29**, 835–847. 10.1118/1.1473134 12033580

[bb19] Cole, A. (1969). *Radiat. Res.* **38**, 7–33. 5777999

[bb20] Collaborative Computational Project, Number 4 (1994). *Acta Cryst.* D**50**, 760–763.

[bb21] Compton, A. H. & Freeman, N. L. (1922). *Nature (London)*, **110**, 38.

[bb22] Coppens, P. (1999). *International Tables for Crystallography*, Vol. *B.*, 2nd ed., ch. 1.2. Dordrecht: Kluwer Academic Publishers.

[bb23] Cork, C., Fehr, D., Hamlin, R., Vernon, W., Xuong, N. H. & Perez-Mendez, V. (1974). *J. Appl. Cryst.* **7**, 319–323.

[bb24] Coster, D. & Kronig, R. de L. (1935). *Physica*, **2**, 13–24.

[bb25] Coulibaly, F., Chiu, E., Ikeda, K., Gutmann, S., Haebel, P. W., Schulze-Briese, C., Mori, H. & Metcalf, P. (2007). *Nature (London)*, **446**, 97–101. 10.1038/nature05628 17330045

[bb26] Cowan, J. A. & Nave, C. (2008). *J. Synchrotron Rad.* **15**, 458–462. 10.1107/S0909049508014623 18728316

[bb27] Creagh, D. C. & Helliwell, J. R. (1999). *International Tables for Crystallography*, Vol. *C*, 2nd ed., ch. 4.2.4. Dordrecht: Kluwer Academic Publishers.

[bb28] Darwin, C. G. (1914). *Philos. Mag.* **27**, 315–333.

[bb29] Darwin, C. G. (1922). *Philos. Mag.* **43**, 800–829.

[bb30] Dauter, Z. (1999). *Acta Cryst.* D**55**, 1703–1717. 10.1107/s0907444999008367 10531520

[bb31] Debye, P. J. W. (1914). *Ann. Phys.* **348**, 49–92.

[bb32] Debye, P. J. W. (1915). *Ann. Phys.* **351**, 809–823.

[bb33] Debye, P. J. W. (1988). *The Collected Papers of Peter J. W. Debye.* Woodbridge: Ox Bow Press.

[bb34] Debye, P. J. W. & Scherrer, P. (1918). *Phys. Z.* **19**, 474–483.

[bb35] Dickerson, R. E., Kendrew, J. C. & Strandberg, B. E. (1961). *Acta Cryst.* **14**, 1188–1195.

[bb36] Drenth, J. (1999). *Principles of Protein X-ray Crystallography.* Berlin: Springer-Verlag.

[bb37] Dwiggins, C. W. (1975). *Acta Cryst.* A**31**, 395–396.

[bb38] Edimo, P., Clermont, C., Kwato, M. G. & Vynckier, S. (2008). *Phys. Med.* **25**, 111–121. 10.1016/j.ejmp.2008.07.001 18722148

[bb39] Einstein, A. (1905). *Ann. Phys.* **322**, 549–560.

[bb40] Ewald, P. P. (1913). *Phys. Z.* **14**, 465–472.

[bb41] Facciotti, M. T., Cheung, V. S., Nguyen, D., Rouhani, S. & Glaeser, R. M. (2003). *Biophys. J.* **85**, 451–458. 10.1016/S0006-3495(03)74490-7 PMC1303101 12829500

[bb42] Flack, H. D. & Vincent, M. G. (1978). *Acta Cryst.* A**34**, 489–491.

[bb43] Garman, E. F. & McSweeney, S. M. (2007). *J. Synchrotron Rad.* **14**, 1–3. 10.1107/S0909049506053015 17211066

[bb44] Garman, E. F. & Nave, C. (2009). *J. Synchrotron Rad.* **16**, 129–132.

[bb45] Garrett, B. C. *et al.* (2004). *Chem. Rev.* **105**, 355–390.

[bb46] Glaeser, R., Facciotti, M., Walian, P., Rouhani, S., Holton, J., MacDowell, A., Celestre, R., Cambie, D. & Padmore, H. (2000). *Biophys. J.* **78**, 3178–3185. 10.1016/S0006-3495(00)76854-8 PMC1300899 10827994

[bb47] Gonzalez, A., Denny, R. & Nave, C. (1994). *Acta Cryst.* D**50**, 276–282. 10.1107/S0907444993013101 15299439

[bb48] Gonzalez, A. & Nave, C. (1994). *Acta Cryst.* D**50**, 874–877. 10.1107/S0907444994006311 15299355

[bb49] Hartree, D. R. (1925). *Philos. Mag. Ser. 6*, **50**, 289–306.

[bb50] Helliwell, J. R. (1999). *International Tables for Crystallography*, Vol. *C*, 2nd ed., ch. 2.2. Dordrecht: Kluwer Academic Publishers.

[bb51] Henderson, R. (1990). *Proc. R. Soc. Lond. B Biol. Sci.* **241**, 6–8.

[bb52] Hendricks, J. S., Adam, K. J., Booth, T. E., Briesmeister, J. F., Carter, L. L., Cox, L. J., Favorite, J. A., Forster, R. A., McKinney, G. W. & Prael, R. E. (2000). *Appl. Radiat. Isot.* **53**, 857–861. 10.1016/s0969-8043(00)00231-1 11003531

[bb53] Holton, J. M. (2009). *J. Synchrotron Rad.* **16**, 133–142.

[bb54] Howells, M. R., Beetz, T., Chapman, H. N., Cui, C., Holton, J. M., Jacobsen, C. J., Kirz, J., Lima, E., Marchesini, S., Miao, H., Sayre, D., Shapiro, D. A., Spence, J. H. C. & Starodub, D. (2009). *J. Electron Spectrosc. Relat. Phenom.* **170**, 4–12. 10.1016/j.elspec.2008.10.008 PMC2867487 20463854

[bb55] Hubbell, J. H. (2006). *Phys. Med. Biol.* **51**, R245–R262. 10.1088/0031-9155/51/13/R15 16790906

[bb56] ICRU (1983). *Microdosimetry.* Report No. 36. Washington, DC: International Commission on Radiological Units and Measurements.

[bb57] James, R. W. (1962). *The Optical Principles of the Diffraction of X-­rays.* London: Bell.

[bb58] Kahn, R., Fourme, R., Gadet, A., Janin, J., Dumas, C. & André, D. (1982). *J. Appl. Cryst.* **15**, 330–337.

[bb59] Kantardjieff, K. A. & Rupp, B. (2003). *Protein Sci.* **12**, 1865. 10.1110/ps.0350503 PMC2323984 12930986

[bb60] Kawrakow, I. & Rogers, D. W. O. (2001). *The EGSnrc Code System: Monte Carlo Simulation of Electron and Photon Transport.* NRCC Report PIRS-701. Ottowa: National Research Council of Canada.

[bb61] Kendrew, J. C., Dickerson, R. E., Strandberg, B. E., Hart, R. G., Davies, D. R., Phillips, D. C. & Shore, V. C. (1960). *Nature (London)*, **185**, 422–427. 10.1038/185422a0 18990802

[bb62] Kmetko, J., Husseini, N. S., Naides, M., Kalinin, Y. & Thorne, R. E. (2006). *Acta Cryst.* D**62**, 1030–1038. 10.1107/S0907444906023869 16929104

[bb63] Kraft, P., Bergamaschi, A., Broennimann, Ch., Dinapoli, R., Eikenberry, E. F., Henrich, B., Johnson, I., Mozzanica, A., Schlepütz, C. M., Willmott, P. R. & Schmitt, B. (2009). *J. Synchrotron Rad.* **16**, 368–375. 10.1107/S0909049509009911 PMC2678015 19395800

[bb64] Leiros, H.-K. S., Timmins, J., Ravelli, R. B. G. & McSweeney, S. M. (2006). *Acta Cryst.* D**62**, 125–132. 10.1107/S0907444905033627 16421442

[bb65] Leslie, A. G. W. (2006). *Acta Cryst.* D**62**, 48–57. 10.1107/S0907444905039107 16369093

[bb66] Li, J., Edwards, P. C., Burghammer, M., Villa, C. & Schertler, G. F. (2004). *J. Mol. Biol.* **343**, 1409–1438. 10.1016/j.jmb.2004.08.090 15491621

[bb67] Lipson, H. & Langford, J. I. (1999). *International Tables for Crystallography*, Vol. *C*, 2nd ed., ch. 6.2. Dordrecht: Kluwer Academic Publishers.

[bb68] MacDowell, A. A. *et al.* (2004). *J. Synchrotron Rad.* **11**, 447–455.

[bb69] Maslen, E. N. (1999). *International Tables for Crystallography*, Vol. *C*, 2nd ed., ch. 6.3. Dordrecht: Kluwer Academic Publishers.

[bb70] Maslen, E. N., Fox, A. G. & O’Keefe, M. A. (1999*a*). *International Tables for Crystallography*, Vol. *C*, 2nd ed., ch. 6.1. Dordrecht: Kluwer Academic Publishers.

[bb71] Maslen, E. N., Fox, A. G. & O’Keefe, M. A. (1999*b*). *International Tables for Crystallography*, Vol. *C*, 2nd ed., Table 6.1.1.4. Dordrecht: Kluwer Academic Publishers.

[bb72] Matthews, B. W. (1968). *J. Mol. Biol.* **33**, 491–497. 10.1016/0022-2836(68)90205-2 5700707

[bb73] Maxwell, J. C. (1865). *Philos. Trans. R. Soc. Lond.* **155**, 459–512.

[bb74] Meitner, L. (1922). *Z. Phys. A*, **9**, 131–144.

[bb75] Moseley, H. G. J. (1913). *Philos. Mag.* **26**, 1024–1034.

[bb76] Moseley, H. G. J. & Darwin, C. G. (1913). *Philos. Mag.* **26**, 210–232.

[bb77] Moukhametzianov, R., Burghammer, M., Edwards, P. C., Petit­demange, S., Popov, D., Fransen, M., McMullan, G., Schertler, G. F. X. & Riekel, C. (2008). *Acta Cryst.* D**64**, 158–166. 10.1107/S090744490705812X PMC2467531 18219115

[bb78] Moussa, H. M., Eckerman, K. F. & Townsend, L. W. (2006). *Radiat. Prot. Dosimetry*, **121**, 252–256. 10.1093/rpd/ncl039 16603605

[bb79] Murray, J. W., Garman, E. F. & Ravelli, R. B. G. (2004). *J. Appl. Cryst.* **37**, 513–522.

[bb80] Murray, J. W., Rudiño-Piñera, E., Owen, R. L., Grininger, M., Ravelli, R. B. G. & Garman, E. F. (2005). *J. Synchrotron Rad.* **12**, 268–275. 10.1107/S0909049505003262 15840910

[bb81] Murshudov, G. N., Vagin, A. A. & Dodson, E. J. (1997). *Acta Cryst.* D**53**, 240–255. 10.1107/S0907444996012255 15299926

[bb82] Murshudov, G. N., Vagin, A. A., Lebedev, A., Wilson, K. S. & Dodson, E. J. (1999). *Acta Cryst.* D**55**, 247–255. 10.1107/S090744499801405X 10089417

[bb83] Nave, C. & Hill, M. A. (2005). *J. Synchrotron Rad.* **12**, 299–303. 10.1107/S0909049505003274 15840914

[bb84] Nelson, R., Sawaya, M. R., Balbirnie, M., Madsen, A. O., Riekel, C., Grothe, R. & Eisenberg, D. (2005). *Nature (London)*, **435**, 773–778. 10.1038/nature03680 PMC1479801 15944695

[bb85] Nelson, W. R., Hirayama, H. & Rogers, D. W. O. (1985). *The EGS4 Code System.* Stanford Linear Accelerator Center Report SLAC-­265.

[bb86] Ott, H. (1935). *Ann. Phys.* **23**, 169–196.

[bb87] Owen, R. L., Holton, J. M., Schulze-Briese, C. & Garman, E. F. (2009). *J. Synchrotron Rad.* **16**, 143–151. 10.1107/S0909049508040429 PMC2651761 19240326

[bb88] Owen, R. L., Rudino-Pinera, E. & Garman, E. F. (2006). *Proc. Natl Acad. Sci. USA*, **103**, 4912–4917. 10.1073/pnas.0600973103 PMC1458769 16549763

[bb89] Paithankar, K. S., Owen, R. L. & Garman, E. F. (2009). *J. Synchrotron Rad.* **16**, 152–162. 10.1107/S0909049508040430 19240327

[bb90] Pflugrath, J. W. (1999). *Acta Cryst.* D**55**, 1718–1725. 10.1107/s090744499900935x 10531521

[bb91] Purcell, E. M. (1985). *Electricity and Magnetism*, 2nd ed. New York: McGraw-Hill.

[bb92] Ramachandran, G. N. & Wooster, W. A. (1951). *Acta Cryst.* **4**, 335–344.

[bb93] Sabine, T. M. (1999). *International Tables for Crystallography*, Vol. *C*, 2nd ed., ch. 6.4. Dordrecht: Kluwer Academic Publishers.

[bb94] Sawaya, M. R., Sambashivan, S., Nelson, R., Ivanova, M. I., Sievers, S. A., Apostol, M. I., Thompson, M. J., Balbirnie, M., Wiltzius, J. J. W., McFarlane, H. T., Madsen, A. O., Riekel, C. & Eisenberg, D. (2007). *Nature (London)*, **447**, 453–457. 10.1038/nature05695 17468747

[bb95] Schulze-Briese, C., Brönnimann, Ch., Eikenberry, E. F., Billich, H., Diez, J., Henrich, B., Kobas, M., Näf, M., Panepucci, E. & Tomizaki, T. (2007). *Acta Cryst.* A**63**, s87.

[bb96] Seltzer, S. M. (1993). *Radiat. Res.* **136**, 147–170. 8248472

[bb97] Shmueli, U. & Wilson, A. J. C. (1999). *International Tables for Crystallography*, Vol. *B*, 2nd ed., ch. 2.1. Dordrecht: Kluwer Academic Publishers.

[bb98] Slater, J. C. (1929). *Phys. Rev.* **34**, 1293.

[bb99] Sliz, P., Harrison, S. C. & Rosenbaum, G. (2003). *Structure*, **11**, 13–19. 10.1016/s0969-2126(02)00910-3 12517336

[bb100] Snell, E. H., Bellamy, H. D. & Borgstahl, G. E. (2003). *Methods Enzymol.* **368**, 268–288. 10.1016/S0076-6879(03)68015-8 14674279

[bb101] Standfuss, J., Xie, G., Edwards, P. C., Burghammer, M., Oprian, D. D. & Schertler, G. F. (2007). *J. Mol. Biol.* **372**, 1179–1188. 10.1016/j.jmb.2007.03.007 PMC2258155 17825322

[bb102] Starodub, D., Rez, P., Hembree, G., Howells, M., Shapiro, D., Chapman, H. N., Fromme, P., Schmidt, K., Weierstall, U., Doak, R. B. & Spence, J. C. H. (2008). *J. Synchrotron Rad.* **15**, 62–73. 10.1107/S0909049507048893 18097080

[bb103] Storm, E. & Israel, H. I. (1970). *Nuclear Data Tables*, **7**, 565–581.

[bb104] Teng, T. & Moffat, K. (2000). *J. Synchrotron Rad.* **7**, 313–317. 10.1107/S0909049500008694 16609214

[bb105] Teng, T.-Y. & Moffat, K. (2002). *J. Synchrotron Rad.* **9**, 198–201. 10.1107/s0909049502008579 12091725

[bb106] Thomson, J. J. (1906). *Conduction of Electricity Through Gases.* Cambridge University Press.

[bb107] Tronrud, D. E. (1997). *Methods Enzymol.* **277**, 306–319. 10.1016/s0076-6879(97)77017-4 9379924

[bb108] Tronrud, D. E. (2007). *Methods Mol. Biol.* **364**, 231–254. 10.1385/1-59745-266-1:231 17172769

[bb109] Waller, I. (1923). *Z. Phys.* **17**, 398–408.

[bb110] Waller, I. (1925). *Theoretische Studien zur Interferenz- und Dispersionstheorie der Röntgenstrahlen.* Dissertation. Uppsala University, Sweden.

[bb111] Welberry, T. R. (2004). *Diffuse X-ray Scattering and Models of Disorder.* Oxford University Press.

[bb112] Wilson, A. J. C. (1942). *Nature (London)*, **150**, 152.

[bb113] Wilson, A. J. C. (1949). *Acta Cryst.* **2**, 318–321.

[bb114] Wilson, A. J. C. & Prince, E. (1999). Editors. *International Tables for Crystallography*, Vol. *C*, 2nd ed. Dordrecht: Kluwer Academic Publishers.

[bb115] Winn, M. D. (2003). *J. Synchrotron Rad.* **10**, 23–25. 10.1107/s0909049502017235 12511787

[bb116] Winn, M. D., Murshudov, G. N. & Papiz, M. Z. (2003). *Methods Enzymol.* **374**, 300–321. 10.1016/S0076-6879(03)74014-2 14696379

[bb117] Woolfson, M. M. (1997). *An Introduction to X-ray Crystallography.* Cambridge University Press.

[bb118] Xuong, N. H., Nielsen, C., Hamlin, R. & Anderson, D. (1985). *J. Appl. Cryst.* **18**, 342–350.

[bb119] Zwart, P. H., Afonine, P. V., Grosse-Kunstleve, R. W., Hung, L.-W., Ioerger, T. R., McCoy, A. J., McKee, E., Moriarty, N. W., Read, R. J., Sacchettini, J. C., Sauter, N. K., Storoni, L. C., Terwilliger, T. C. & Adams, P. D. (2008). *Methods Mol. Biol.* **426**, 419–435. 10.1007/978-1-60327-058-8_28 18542881

