# Supplementary material for: The minimum crystal size needed for a complete diffraction data set
Source: Acta Crystallogr D Biol Crystallogr. 2010 Mar 24;66(Pt 4):393–408. doi: 10.1107/S0907444910007262 (PMC2852304; doi:10.1107/S0907444910007262)
Supplement: Supplementary file 1 [file d-66-00393-sup1.pdf]

Appendix A: Data completeness

The fraction of relps in a given constant-resolution sphere that are lost in the blind region lie in two polar caps intersecting the rotation axis (z). That is, all points in reciprocal space that share a common d-spacing form a sphere, and as this constant-resolution sphere is "spun" about the z-axis, most points will cross the Ewald sphere twice, some not at all (near the axis), and a few will intersect it only once. These single-hit relps "graze" the Ewald sphere, and form the border between the two-hit and zero-hit regions. The "grazing relp" border is a circle, and it is illustrative to consider moving the relp circle in Fig. 1 up the z axis until it intersects the Ewald sphere at just one point. At this grazing point, Bragg's Law:

(A1) $\lambda = 2d \sin\theta$

is satisfied, and so the height (h) of this circle above the $z = 0$ plane must be the radius of the Ewald sphere λ^* times the sin of the take-off angle of the spot (2θ):

(A2) $h = \lambda^* \sin 2\theta$

Now, the area in either of the blind regions is a section of the surface of a sphere that is cut off by a plane at $z = \pm h$. This shape is known as a spherical cap, and the area of a spherical cap from a sphere of radius r that was cut at height h is given by:

(A3) $A_{cap} = 2\pi\, r\, (r-h)$

There are two such caps, and we are interested in the area left over after they are cut off (observable relps) relative to the original area of the sphere:

(A4) $$\mathrm{f}_{obs} = \frac{4\pi r^2 - 2\left(2\pi r\left(r\text{-}h\right)\right)}{4\pi r^2} = \frac{r-\left(r-h\right)}{r} = \frac{h}{r}$$

Since a sphere of relps with constant d-spacing "*d*" has radius $1/d = d^*$, we may substitute $r = d^*$ and Equation (A2) into Equation (A4):

$$\text{(A5)} \quad \mathrm{f}_{obs} = \frac{\lambda^* \sin 2\theta}{d^*} = \frac{d}{\lambda} 2\sin\theta\cos\theta$$

Substituting in Equation (A1) for λ:

$$\text{(A6)} \quad \mathrm{f}_{obs} = \frac{d}{2d\sin\theta} 2\sin\theta\cos\theta = \cos\theta$$

Appendix B: Average Lorentz-polarization factor

Taking the product of Equations (2a) and (3) from the text and, for the moment, simplifying Equation (3) by considering the case of an unpolarized beam ($\mathscr{I} = 0$), we have:

$$\text{(B1)} \quad LP = \frac{1+\cos^2 2\theta}{2\sqrt{\sin^2 2\theta - \zeta^2}}$$

Assuming the X-ray beam is perpendicular to the rotation axis, the polar coordinate ζ is simply the height of the relp circle pictured in Fig. 1 above the $z = 0$ plane if the length of the relp vector is normalized to unit wavelength. That is, if we define the angle κ between the relp vector and the $z = 0$ plane, we have:

$$\text{(B2)} \quad \zeta = \lambda \left| \mathbf{d}^* \right| \sin\kappa = \frac{\lambda \sin\kappa}{d} = 2 \sin\theta \sin\kappa$$

The angle κ is 0° when the relp circle lies exactly in the $z = 0$ plane, and it is 90° when the relp lies perfectly along the z-axis and the relp circle has vanished. Note that Equation (B1) is undefined at this position, and so care must be taken when approaching

this singularity. The value of ζ for which the relp circle just grazes the Ewald sphere at one point is when the denominator of (B1) becomes zero, or:

(B3) $\quad \sin 2\theta = \zeta = 2\ \sin\theta\ \sin\kappa \quad \text{or} \quad \sin\kappa = \cos\theta$

Since we wish to avoid the singularity here, we shall set our limit of integration at a small value short of it and use the symbol η because it is reminiscent of mosaic spread.

(B4) $\quad \kappa_{\max} = \arcsin(\cos\theta) - \eta$

Substituting B2 into B1 we obtain:

(B5) $$LP = \frac{1+\cos^2 2\theta}{2\sqrt{\sin^2 2\theta - 4\sin^2\theta\ \sin^2\kappa}} = \frac{1+\cos^2 2\theta}{4\sin\theta\sqrt{\cos^2\theta -\ \sin^2\kappa}}$$

Computing the average value of *LP* for all spots at a fixed resolution is equivalent to integrating *LP* over the accessible surface of the constant-resolution sphere, and then dividing by the accessible surface area:

(B6) $$\langle LP \rangle = \frac{\int_{equator}^{blind} LP\ dA}{\int_{equator}^{blind} dA}$$

The denominator of (B6) was solved as Equation (A6), and is simply half of the accessible surface area of the constant-resolution sphere, which has radius λ/d or $2\sin\theta$, and accessible fraction $f_{obs} = \cos\theta$:

(B7) $$\int_{equator}^{blind} dA = 2\ \pi(2\sin\theta)^2\ \cos\theta$$

Now, all the points that lie on or very near the relp circle pictured in Fig. 1 will have the same *L* and *P* factors, so we must "weight" the *LP* of each contact point on the Ewald sphere surface by the circumference of the relp circle. That is, we define the area element

in Equation (B6) with a re-casting of Equation (A3) where we can compute the area of the constant-resolution sphere above the relp circle in Fig. 1 by substituting the radius of this sphere ($\lambda/d = 2\sin\theta$) for "*r*" and ζ from Equation (B2) for "*h*":

(B8) $$A_{cap} = 2\pi(2\sin\theta)(2\sin\theta - 2\sin\theta\sin\kappa) = 8\pi\sin^2\theta - 8\pi\sin^2\theta\sin\kappa$$

Differentiating with respect to κ, we obtain the area element:

(B9) $$dA = -8\pi\sin^2\theta\cos\kappa\, d\kappa$$

Substituting (B5), (B7) and (B9) into (B6) we have:

(B10a) $$\langle LP\rangle = \frac{\int_0^{\kappa_{\max}} \frac{1+\cos^2 2\theta}{4\sin\theta\sqrt{\cos^2\theta - \sin^2\kappa}}\left(-8\pi\sin^2\theta\cos\kappa\right) d\kappa}{2\pi(2\sin\theta)^2\cos\theta}$$

(B10b) $$\langle LP\rangle = -\frac{1+\cos^2 2\theta}{4\sin\theta\cos\theta}\int_0^{\kappa_{\max}} \frac{\cos\kappa}{\sqrt{\cos^2\theta - \sin^2\kappa}}\, d\kappa$$

We now employ the indefinite integral:

(B11) $$\int \frac{\cos\kappa}{\sqrt{C - \sin^2\kappa}}\, d\kappa = -\arctan\left(\frac{\sin^2\kappa\sqrt{C - \sin^2\kappa}}{\sin^2\kappa - C}\right)$$

And substitute this into the definite integral:

(B12)

$$\int_0^{\kappa_{\max}} \frac{\cos\kappa}{\sqrt{\cos^2\theta - \sin^2\kappa}}\, d\kappa = -\arctan\left(\frac{(\cos^2\theta - \eta)\sqrt{\cos^2\theta - (\cos^2\theta - \eta)}}{(\cos^2\theta - \eta) - \cos^2\theta}\right) + \arctan\left(\frac{(0)\sqrt{\cos^2\theta - (0)}}{(0) - \cos^2\theta}\right)$$

(B13) $$\int_0^{\kappa_{\max}} \frac{\cos\kappa}{\sqrt{\cos^2\theta - \sin^2\kappa}}\, d\kappa = -\arctan\left(\frac{(\cos^2\theta - \eta)\sqrt{\eta}}{-\eta}\right)$$

Clearly, as we approach the limit:

$$\text{(B14)} \quad \lim_{\eta \to 0} \int_0^{\kappa_{\max}} \frac{\cos\kappa}{\sqrt{\cos^2\theta - \sin^2\kappa}} d\kappa = \frac{-\pi}{2}$$

Substituting this back into Equation (B10b) we obtain the average *LP* factor:

$$\text{(6a)} \quad \langle LP \rangle = \frac{\pi}{8} \frac{1+\cos^2 2\theta}{\sin\theta\cos\theta} = \frac{\pi}{2}\left(\frac{1}{\sin 2\theta} - \frac{\sin 2\theta}{2}\right)$$

Multiplying ⟨LP⟩ by $f_{obs} = \cos\theta$, we obtain:

$$\text{(6b)} \quad \langle LP \rangle f_{obs} = \frac{\pi}{8} \frac{1+\cos^2 2\theta}{\sin\theta} = \frac{\pi(3+\cos 4\theta)}{16\sin\theta}$$

This result was validated numerically by calculating discrete spot positions, *L* and *P* for hypothetical data sets using randomized wavelengths, unit cells and crystal orientations and then dividing the predictions into resolution bins and averaging the value of the product *LP* and the fraction of all possible relps that appeared in the bin (not shown). These simulations were repeated using the full expression for the polarization factor with different values for the degree of polarization ($\mathscr{I}$), but the numerical results were identical to $\mathscr{I} = 0$ (not shown), and we saw no need to repeat the derivation using the full polarization factor expression.

Appendix C: Spot-fading integral

Here we assume that the average spot intensity (photons/spot) at a given resolution fades exponentially, but begin with a slightly different representation of Equation (13) than in the text:

(C1) $\langle i \rangle(t) = \langle i \rangle(0)\ \exp\left(-\ln(2)\dfrac{DR}{H\ d}t\right)$

where:

$\langle i \rangle$ (t) - average intensity rate (photons/spot/s) at time t (after absorbing a dose $DR \cdot t$)

$\langle i \rangle$ (0) - average intensity rate (photons/spot/s) from an undamaged crystal

ln(2) - natural log of two (~0.7)

DR - dose deposited per unit time (D_{reso}/t) or dose rate (MGy/s)

H - Howells *et al.* (2009) criterion (10 MGy/Å)

d - d-spacing (Å)

t - accumulated exposure time (seconds)

Note the use of angle brackets $\langle\ \rangle$ to denote the average in a given resolution bin, and that here we use the term "intensity rate" to refer to a spot intensity (photons/spot) divided by the exposure time used to record it (seconds). This is because accurate spot fading experiments must record the decay curve by sampling the same spots over and over again using per-observation exposure times that are short relative to the damage limit (T_{DL}). That is, when sampling a changing signal, the sampling time must be short enough so that the signal does not decay appreciably during a given sample. This is equivalent to requiring that the photons/spot per unit time (intensity rate) is constant for a given exposure. So, effectively, spot fading experiments measure changes in intensity rate. Here we use a lower case "*i*" to differentiate an intensity rate (photons/spot/s) from an integrated spot intensity (photons/spot), which we will continue to denote with a capital "*I*", and also replace the dose D_{reso} with $DR \times t$ so that *DR* represents the time-invariant factors of dose in Equation (11). Since *DR, d, H*, and $\langle i \rangle$ (0) do not change with time, the integral of Equation (C1) is simply the integral of an exponential decay:

$$\text{(C2)} \quad \langle I \rangle_{DL} = \int_0^{T_{DL}} \langle i \rangle(t)\, dt = \langle i \rangle(0) \frac{H\ d}{\ln(2)\ DR}\left(\exp(0) - \exp\left(-\frac{\ln(2)\ DR}{H\ d} T_{DL} \right)\right)$$

Where:

$\langle I \rangle_{DL}$ - accumulated spot intensity at the damage limit (photons/spot)

T_{DL} - accumulated exposure time at the damage limit (seconds)

DR - dose rate (MGy/s)

H - Howells *et al.* (2009) criterion (10 MGy/Å)

d - d-spacing in Å

Since we are not considering the accumulation of background counts, T_{DL} could be chosen to be infinity and $\langle I \rangle_{DL}$ would then truly account for every last photon that will fall into a spot before it fades away completely, but in practice the damage limit is usually declared at a point where $\langle i \rangle (T_{DL})$ is not zero, as discussed in §2.11. To account for potentially variable damage limit criteria, we define the "decay fraction" at the end of data collection as:

$$\text{(C3)} \quad \mathrm{f}_{decayed} = \frac{\langle i \rangle(0) - \langle i \rangle(T_{DL})}{\langle i \rangle(0)}$$

Since T_{DL} is now defined in terms of $\mathrm{f}_{decayed}$, substituting Equation (C3) into Equation (C2) simplifies it to the expression:

$$\text{(C4)} \quad \langle I \rangle_{DL} = \langle i \rangle(0)\ \mathrm{f}_{decayed} \frac{H\ d}{\ln(2)\ DR}$$

where:

$\langle I \rangle_{DL}$ - average accumulated spot intensity (photons/spot) at the damage limit

$\langle i \rangle (0)$ - average intensity rate (photons/spot/s) from an undamaged crystal

DR - dose rate (MGy/s)

H - Howells *et al.* (2009) criterion (10 MGy/Å)

d - d-spacing in Å

Although it may appear that $\langle I \rangle_{DL}$ (photons/spot) depends on the dose rate (*DR*), the time component of *DR* (MGy/s) is actually cancelled by the time component of the initial intensity rate $\langle i \rangle(0)$ (photons/spot/s). Consider a hypothetical data collection strategy where a very large number of observations are made of each spot, and the whole data set is actually a series of "mini" data sets with exposure time t_{DS}. As long as t_{DS} is very small when compared to the damage limit (T_{DL}), the spot intensities (photons/spot) in the first "mini" data set (I_{ND}) will be "undamaged" and given by Darwin's formula (Equation (1)). The exact I_{ND} measured will be proportional to t_{DS}, as can easily be seen by substituting ω_{eff} from Equation (10) into Equation (1):

(C5) $$\frac{I_{ND}}{t_{DS}} = I_{beam} r_e^2 \frac{V_{xtal}}{V_{cell}} \cdot \frac{4 n_{symop}}{2\pi} \frac{\lambda^3}{V_{cell}} LP \cdot A \cdot |F|^2$$

where:

I_{ND} - spot intensity (photons/spot) from the first "mini" data set measured quickly enough to have suffered no radiation damage effects.

It is readily apparent that the quotient I_{ND}/t_{DS} (photons/spot/s) is an intensity rate, as is the average value $\langle I \rangle_{ND}/t_{DS}$. Formally, the right hand side of Equation (C5) is independent of time, but as the reality of radiation damage progresses the same t_{DS} will record spot intensities that fade (on average) according to the exponential decay of Equation (C1). Therefore, the starting value of this decay curve is $\langle I \rangle_{ND}/t_{DS}$, and we may substitute this intensity rate for $\langle i \rangle(0)$ in the above Equations:

(C6) $\langle i\rangle(0)=\frac{I_{ND}}{t_{DS}}$

Now the sum of all the equivalent observations in all the mini data sets up to a given accumulated exposure time is given by Equation (C2), and even if a single data set were collected with total exposure time T_{DL}, the average number of photons that eventually contribute to a spot ($\langle I\rangle_{DL}$) is simply the integral of the decay over time (Equation (C4)).

We now substitute $\langle I\rangle_{ND}/t_{DS}$ for $\langle i\rangle(0)$ in Equation (C4), as well as D_{en}/t from Equation (11) for the dose rate (DR), convert the photon energy into wavelength ($q_e E_{ph} = hc/\lambda$ = J/photon), apply the Nave-Hill fraction f_{NH} from Equation (12) and scale to convenient units. We arrive at Equation (14) from the main text:

(14) $$\langle I\rangle_{DL}=\frac{\langle I\rangle_{ND}}{t_{DS}}\frac{0.1\ f_{decayed}\ 4\ H\ d\ \lambda\ R\ \rho}{3\ \ln(2)\ f_{NH}\ h\ c\ I_{beam}\left(1-T_{sphere}(0,\mu_{en},R)\right)}$$

Where:

$\langle I\rangle_{DL}$ - maximum average spot intensity due to radiation damage limits (photons/spot)

$\langle I\rangle_{ND}$ - average spot intensity (photons/spot) observed using an undamaged crystal and a very short exposure: t_{DS}

H - Howells's criterion (10 MGy/Å)

0.1 - converting λ from Å to m, ρ from g/cm^3 to kg/m^3 and MGy to Gy

λ - X-ray wavelength (Å)

h - Planck's constant (6.626×10^{-34} J·s)

c - speed of light (299792458 m/s)

R - radius of the spherical crystal (m)

ρ - density of crystal (~1.2 g/cm^3)

I_{beam} - incident beam intensity ($photons/s/m^2$)

μ_{en} - mass energy-absorption coefficient of sphere material (m^{-1})

f_{NH} - the Nave-Hill fraction

References

Howells, M. R., Beetz, T., Chapman, H. N., Cui, C., Holton, J. M., Jacobsen, C. J., Kirz, J., Lima, E., Marchesini, S., Miao, H., Sayre, D., Shapiro, D. A., Spence, J. H. C. & Starodub, D. (2009). *J. Electron Spectrosc. Relat. Phenom.* **170**, 4-12.
